# SR proteins are NXF1 adaptors that link alternative RNA processing to mRNA export

**DOI:** 10.1101/gad.276477.115

**Published:** 2016-03-01

**Authors:** Michaela Müller-McNicoll, Valentina Botti, Antonio M. de Jesus Domingues, Holger Brandl, Oliver D. Schwich, Michaela C. Steiner, Tomaz Curk, Ina Poser, Kathi Zarnack, Karla M. Neugebauer

**Affiliations:** 1 RNA Regulation Group, Institute of Cell Biology and Neuroscience, Goethe-University Frankfurt, 60438 Frankfurt/Main, Germany;; 2 Department of Molecular Biophysics and Biochemistry, Yale University, New Haven, Connecticut 06520, USA;; 3 Institute of Molecular Biology (IMB), 55128 Mainz, Germany;; 4 Max Planck Institute of Molecular Cell Biology and Genetics, 01307 Dresden, Germany;; 5 Buchmann Institute for Life Sciences (BMLS), 60438 Frankfurt/Main, Germany;; 6 Faculty of Computer and Information Science, University of Ljubljana, Ljubljana 1000, Slovenia

**Keywords:** iCLIP, mRNA export, alternative 3′ end processing, SR protein, NXF1, SRSF3, SRSF7

## Abstract

Nuclear export factor 1 (NXF1) exports mRNA to the cytoplasm after recruitment to mRNA by specific adaptor proteins. How and why cells use numerous different export adaptors is poorly understood. Here we critically evaluate members of the SR protein family (SRSF1–7) for their potential to act as NXF1 adaptors that couple pre-mRNA processing to mRNA export. Consistent with this proposal, >1000 endogenous mRNAs required individual SR proteins for nuclear export in vivo. To address the mechanism, transcriptome-wide RNA-binding profiles of NXF1 and SRSF1–7 were determined in parallel by individual-nucleotide-resolution UV cross-linking and immunoprecipitation (iCLIP). Quantitative comparisons of RNA-binding sites showed that NXF1 and SR proteins bind mRNA targets at adjacent sites, indicative of cobinding. SRSF3 emerged as the most potent NXF1 adaptor, conferring sequence specificity to RNA binding by NXF1 in last exons. Interestingly, SRSF3 and SRSF7 were shown to bind different sites in last exons and regulate 3′ untranslated region length in an opposing manner. Both SRSF3 and SRSF7 promoted NXF1 recruitment to mRNA. Thus, SRSF3 and SRSF7 couple alternative splicing and polyadenylation to NXF1-mediated mRNA export, thereby controlling the cytoplasmic abundance of transcripts with alternative 3′ ends.

## Abstract (precis)

In this study, Müller-McNicoll et al. investigate how export machinery assembles on mRNA and how it senses mRNA maturity before exporting mRNAs from the nucleus. They show that SR proteins act as NXF1 adaptors by connecting alternative splicing and 3′ end formation to mRNA export in vivo and propose that SR proteins and NXF1 form a ternary complex on mRNAs, particularly in last exons, and shuttle together to the cytoplasm.

Export of mRNA from the nucleus to the cytoplasm is a highly regulated step in gene expression. Export is mediated by nuclear export factor 1 (NXF1), which contains two distinct RNA-binding domains (RBDs) that are both essential for mRNA export (Braun et al. 2001; Hautbergue et al. 2008). The pseudo-RNA recognition motif (ψRRM) and the adjacent leucine-rich region constitute one RBD, which binds exclusively to constitutive transport elements (CTEs) of retroviruses with high affinity and sequence specificity (Teplova et al. 2011). In contrast, the second RBD of NXF1 at the N terminus is an arginine-rich region that binds RNA without sequence specificity. Therefore, NXF1 relies on different adaptor proteins to regulate export of mature cellular mRNAs (Walsh et al. 2010; Viphakone et al. 2012).

The prevailing model for adaptor function holds that free NXF1 forms a closed loop in which the arginine-rich RBD is hidden and RNA binding is inhibited. Coordinated binding of adaptors triggers a conformational switch in NXF1 that opens the loop, exposes the RBD, and enables NXF1 to bind to mRNA (Viphakone et al. 2012). The model is based on studies of previously characterized NXF1 adaptor proteins, including RBM15, UIF, CHTOP, and LUZP4 as well as ALYREF, THOC5, and UAP56 of the transcription export complex (TREX) (Huang et al. 2003; Taniguchi and Ohno 2008; Hautbergue et al. 2009; Katahira et al. 2009; Uranishi et al. 2009; Walsh et al. 2010; Viphakone et al. 2015). Most of these adaptors recruit NXF1 to pre-mRNA 5′ ends via the cap-binding complex (CBC) (Cheng et al. 2006; Nojima et al. 2007), ensuring that exported mRNAs are capped and routing them toward the NXF1 export pathway (Müller-McNicoll and Neugebauer 2013). For example, ALYREF and THOC5 ensure that NXF1 is loaded onto pre-mRNA only when both adaptors are assembled within the TREX complex, which occurs cotranscriptionally (Masuda et al. 2005; Viphakone et al. 2012). Therefore, the TREX family of adaptors couple transcription and mRNA 5′ end capping to export. However, NXF1 can export unspliced pre-mRNA efficiently to the cytoplasm when tethered or directly bound to it (Hargous et al. 2006; Li et al. 2006), raising the important question of how export mechanisms sense mRNA maturity. Perhaps the great diversity among NXF1 adaptors provides the cell with the potential to couple mRNA export to additional steps in mRNA processing, such as splicing and 3′ end formation.

SR proteins are essential RNA-binding proteins (RBPs) with evolutionarily conserved roles as regulators of constitutive and alternative pre-mRNA splicing (Änkö 2014; Howard and Sanford 2015). SR proteins regulate such diverse processes as 3′ end processing (Lou et al. 1998; Bradley et al. 2015), mRNA export (Masuyama et al. 2004; Huang and Steitz 2005), mRNP packaging (Singh et al. 2012), mRNA stability (Lemaire et al. 2002), and translation (Michlewski et al. 2008; Maslon et al. 2014). They are recruited to pre-mRNA during transcription, consistent with cotranscriptional assembly of the spliceosome and splicing (Sapra et al. 2009) and suggesting that SR proteins may couple sequential events and mark mRNAs as they transit from the nucleus to the cytoplasm.

The SR protein family comprises seven canonical members (SRSF1 to SRSF7) that are structurally related but functionally distinct. All family members contain one or two RRMs at their N termini. The number and spacing of the RRMs as well as their combination with additional domains confer substrate specificity, resulting in divergent RNA-binding preferences in vivo (Clery et al. 2008). So far, in vivo binding motifs have been determined for four mammalian SR proteins (Sanford et al. 2008; Änkö et al. 2012; Pandit et al. 2013). At their C termini, SR proteins possess regions of repeated serine–arginine dipeptides (RS domains) that may mediate protein–protein and/or RNA–protein interactions (Shen et al. 2004). Extensive serine phosphorylation within the RS domain is crucial for SR protein recruitment to transcription sites and for spliceosome assembly; RS domain dephosphorylation occurs during splicing and is important for catalysis, release of the splicing machinery, and subsequent mRNP maturation (Huang and Steitz 2005; Shepard and Hertel 2009; Ghosh and Adams 2011).

Several properties of SR proteins suggest that they act as adaptors for NXF1-dependent mRNA export and potentially couple the completion of splicing to mRNA export. SRSF1, SRSF3, and SRSF7 bind directly to NXF1 only in their hypophosphorylated state (Lai and Tarn 2004; Huang and Steitz 2005; Hargous et al. 2006; Tintaru et al. 2007), implying that binding occurs after splicing is completed. Moreover, a cycle of RS domain phosphorylation, dephosphorylation, and rephosphorylation is important for the nucleo–cytoplasmic shuttling of SR proteins (Caceres et al. 1998; Cazalla et al. 2002; Huang and Steitz 2005; Lin et al. 2005). SRSF3 and SRSF7 were shown to be required for the export of reporter transcripts containing an export element from the coding region of the histone *H2A* gene (Huang and Steitz 2001). However, replication-dependent histone mRNAs do not contain introns and are not spliced or polyadenylated. Surprisingly little is known about the nuclear export of spliced mRNAs via SR proteins.

Here we set out to determine whether and how different SR protein family members participate in mRNA export in vivo*.* We employed a series of quantitative in vivo experimental approaches to probe the functions of individual SR protein family members (SRSF1–7) in mRNA export using pluripotent mouse P19 cells. First, depletion of individual SR proteins followed by cellular fractionation and RNA sequencing (RNA-seq) led to the identification of endogenous transcripts dependent on SR proteins for export. We conducted individual-nucleotide-resolution UV cross-linking and immunoprecipitation (iCLIP) experiments to globally identify RNA-binding profiles of each SR protein and NXF1 in vivo, enabling us to determine the proximity of NXF1 and SR protein-binding sites and the contribution of SR proteins to NXF1 RNA-binding specificity. Related to this, we quantified SR protein-mediated NXF1 recruitment to endogenous mRNAs. Additional analyses and validation experiments show that SR proteins promote mRNA export of alternatively processed transcripts by recruiting NXF1 to adjacent regulatory sites, suggesting that they shuttle together with mRNA cargo to the cytoplasm.

## Results

### Identification of endogenous mRNA export targets of SR proteins

As NXF1 adaptors, SR proteins are expected to promote the export of specific mRNAs. Our previous work in P19 cells showed that individual SR proteins interact with distinct sets of mRNAs (Änkö et al. 2010), suggesting that specific mRNAs may be controlled by SR protein family members independently (Björk et al. 2009; Pandit et al. 2013; Bradley et al. 2015). Depletion of export adaptors usually causes only a modest export block due to functional substitution (Hautbergue et al. 2009; Katahira et al. 2009; Uranishi et al. 2009). Nevertheless, specific export targets of THO/TREX components have been identified using knockdown and cell fractionation approaches (Rehwinkel et al. 2004; Katahira et al. 2009; Guria et al. 2011). To test whether decreased cytoplasmic transcript levels are a good proxy for export defects in P19 cells, NXF1 was depleted by RNAi, and changes in mRNA levels in cytoplasmic and nuclear fractions were quantified (Supplemental Fig. S1A,B). Upon NXF1 depletion, thousands of transcripts specifically decreased in the cytoplasm, and the corresponding transcripts increased in the nucleus (Supplemental Fig. S1A). We conclude that depletion of mRNA export factors followed by transcriptome analysis of cytoplasmic and total fractions reveals mRNA export targets in our system.

**Figure 1.**
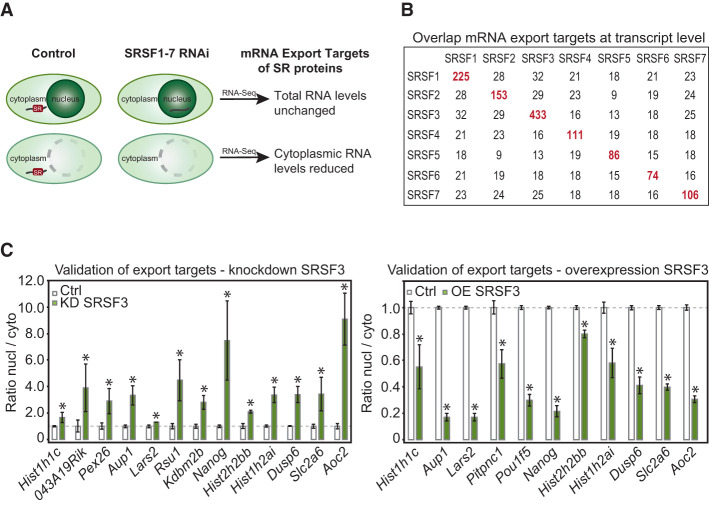
Identification of endogenous mRNA export targets of SR proteins. (*A*) Scheme illustrating the extraction of candidate mRNA export targets from cytoplasmic and whole-cell RNA-seq data after 36 h of SR protein depletion by RNAi. (*B*) Numbers of mRNA export targets for each SR protein and overlaps among them. (*C*) Validation of SRSF3 export targets by RT-qPCR. (*Left* panel) Knockdown (KD) of SRSF3 in P19 wild-type cells leads to an increased nuclear/cytoplasmic ratio of each mRNA. *n* = 5. (*Right* panel) Overexpression (OE) leads to a decreased nuclear/cytoplasmic ratio of each mRNA. *n* = 3. (*) *P* < 0.05.

To determine mRNA targets of SR proteins, individual family members were depleted by RNAi (Supplemental Fig. S1C). RNA-seq libraries were then prepared from whole-cell and cytoplasmic poly(A)^+^ RNA (Fig. 1A; Supplemental Table S1). Transcript levels were highly reproducible between replicates, supporting consistent yield in our fractionations (Supplemental Fig. S1D). Expression changes at the transcript level were quantified using Cuffdiff (Trapnell et al. 2013). In line with previous findings (Änkö et al. 2010; Pandit et al. 2013; Bradley et al. 2015), depletion of SR proteins had positive and negative effects on transcript expression that were validated in every case tested (Supplemental Fig. S1E,F).

To identify endogenous export targets, cytoplasmic mRNA abundance was normalized to whole-cell transcript levels to identify changes in export independent of other alterations in gene expression (Fig. 1A). This yielded a total of 1189 candidate mRNA export targets that exhibited a net decrease in cytoplasmic abundance when individual SR proteins were limiting (Fig. 1B). Each set of export targets was enriched for protein-coding genes (Supplemental Fig. S1G). SRSF3 had the highest number of candidate mRNA export targets (433), and very little overlap was seen among targets of individual SR proteins (Fig. 1B). To validate our experimental system and data analysis, cell fractionation and RT-qPCR of SRSF3 export candidates were conducted after knockdown or overexpression of SRSF3 (Fig. 1C; Supplemental Fig. S1H). Notably, SRSF3 knockdown decreased and overexpression increased cytoplasmic levels of all export candidates tested, in agreement with the proposal that SR proteins promote nuclear export. We conclude that >1000 specific mRNAs are export targets of individual SR proteins.

### SR proteins interact with NXF1 and promote its recruitment to mRNA in vivo

**Figure 2.**
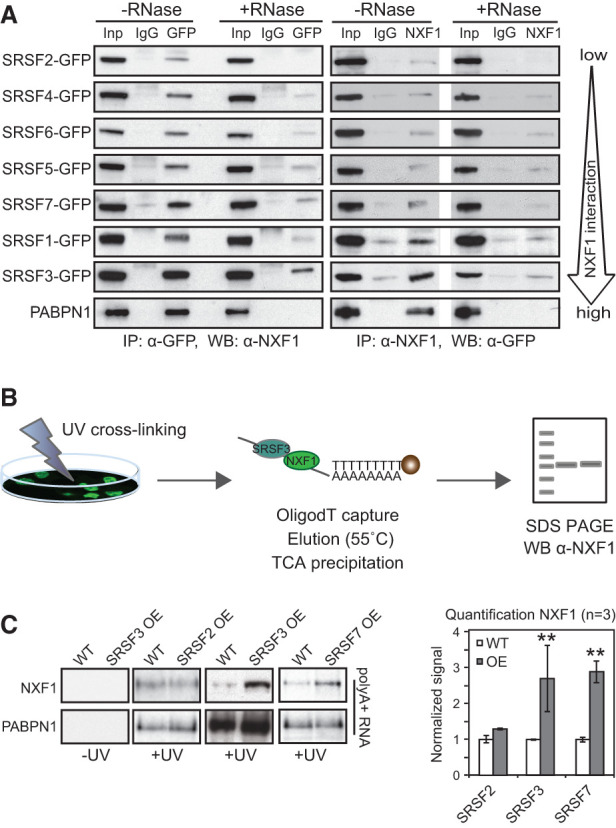
Differential association of SR proteins with NXF1 and promotion of NXF1 binding to mRNA. (*A*) Semiquantitative co-IPs. GFP-tagged SR proteins were pulled down by α-GFP antibodies and probed for interacting NXF1 using α-NXF1 antibodies (forward). Endogenous NXF1 was pulled down using α-NXF1 antibodies and probed for GFP expression (reverse). Lanes marked +RNase show co-IPs carried out after RNase A treatment. PABPN1, which binds polyA tails, served to control successful RNA degradation. (*B*) Scheme of oligodT capture: P19 cells were UV cross-linked, and mRNA was purified using oligodT beads and probed for NXF1 by Western blot (WB). Stringent conditions were used to strip off non-cross-linked proteins (Castello et al. 2012). (*C*, *left* panel) P19 clonal cell lines, selected for high GFP expression by FACS, overexpress SRSF2, SRSF3, or SRSF7 by ∼2.5-fold compared with wild-type (WT) P19 cells (Supplemental Fig. S2F,G). Overexpression (OE) of SRSF2 was quantitated by RT-qPCR because available anti-SRSF2 antibodies were unspecific in our hands. PABPN1 binding served as a loading control. (*Right* panel) Quantification of NXF1 cross-linking to mRNAs upon SRSF2, SRSF3, and SRSF7 overexpression is expressed relative to wild-type. Mean and SD are shown. *n* = 3 independent experiments. (**) *P* < 0.001.

Intriguingly, candidate export targets were detected for all SR proteins, prompting us to compare their interactions with NXF1 in vivo. To do so, we generated stable P19 cell lines expressing physiological levels of functional GFP-tagged SR proteins (SRSF1–7) from recombineered bacterial artificial chromosomes (BACs) (Supplemental Fig. S2A–C; Sapra et al. 2009; Änkö et al. 2010, 2012). We performed semiquantitative forward and reverse coimmunoprecipitations (co-IPs) with antibodies specific for GFP and endogenous NXF1 and found that all SR proteins were detected in NXF1-containing mRNPs (Fig. 2A). RNase A digestion abolished SR protein and NXF1 interactions with PABPN1, which binds polyA tails and is not known to interact with SR proteins or NXF1. Similarly, NXF1 interactions with SRSF2 were completely lost upon RNase A digestion, indicating indirect linkage through RNA (Fig. 2A). In contrast, partial RNase A resistance was consistent with protein–protein interactions between NXF1 and all other SR proteins (Fig. 2A; Supplemental Fig. S2D). Quantification of SR protein–NXF1 interactions from six forward and four reverse co-IP experiments revealed that SRSF3 interacts most robustly with NXF1 in the presence or absence of RNase (Supplemental Fig. S2D).

Although SRSF1, SRSF3, and SRSF7 interact directly with NXF1 in vitro (Huang et al. 2003, 2004; Lai and Tarn 2004; Hargous et al. 2006; Tintaru et al. 2007), the observed RNase sensitivity suggests that RNA binding may stabilize the complex formed in vivo. Indeed, prolonged RNase A treatment abolished NXF1 interactions with SR proteins completely (Supplemental Fig. S2E). Time-course experiments with different incubation times of RNase A showed that NXF1–SRSF1 interactions were maintained at time points long after which rRNAs were completely degraded and the nuclear polyA-binding protein PABPN1 was lost from mRNPs (Supplemental Fig. S2F). The loss of NXF1 binding after 40 min of digestion was attributable to RNA degradation because SRSF1 remained associated with the SR protein kinase 1 (SRPK1) at all time points. These data suggest that NXF1 binding to SRSF1 and likely other SR proteins is stabilized by their assembly on mRNA.

If SR proteins act as genuine NXF1 adaptors, their overexpression should promote NXF1 recruitment to mRNA, as do THO/TREX adaptors (Tintaru et al. 2007; Hautbergue et al. 2008; Viphakone et al. 2012)*.* To determine the amount of NXF1 protein associated with polyA^+^ RNA with and without SR protein overexpression, P19 cells were UV cross-linked and subjected to oligodT capture (Fig. 2B). Quantification revealed that twofold to 2.5-fold overexpression of SRSF3-GFP and SRSF7-GFP significantly enhanced cross-linking of NXF1 to mRNA by 2.5-fold to threefold (Fig. 2C; Supplemental Fig. S2G). Overexpression of SRSF2-GFP, which showed no direct interaction with NXF1, did not enhance NXF1 recruitment to mRNA. These data further suggest that SRSF2 does not serve as an NXF1 adaptor in vivo but rather contributes to export indirectly. In contrast, SRSF3 and SRSF7 interact with NXF1 and promote NXF1 binding to mature mRNA in vivo.

### SR proteins and NXF1 cross-link to spliced mRNAs and reside in mature mRNPs

If SR proteins serve as genuine export adaptors for the mRNA export targets identified above (see Fig. 1), we would expect them to bind directly to these specific targets and recruit NXF1 nearby. To test this, we performed iCLIP with GFP-tagged SRSF1–7 or NXF1-GFP in P19 cells (Supplemental Fig. S3A–C). GFP fused to a nuclear localization sequence (GFP-NLS) served as a negative control (Änkö et al. 2012). Using the same anti-GFP antibody and identical conditions permitted direct comparisons between iCLIP data sets. Biological replicates were highly reproducible (Supplemental Table S2), and pooled reads yielded between 1.8 million and 14 million unique cross-link events per data set (Supplemental Table S3). Significant cross-link events (false discovery rate [FDR] < 0.05) were extracted as previously described (Yeo et al. 2009; König et al. 2010; Wang et al. 2010), yielding a total of 125,474–214,306 binding sites per protein. Each protein cross-linked to thousands of exons and bound similar numbers of target mRNAs across the range of mRNA expression levels (Supplemental Fig. S3D–F). Consistent with previous reports (Änkö et al. 2012; Pandit et al. 2013; Bradley et al. 2015), the majority (90%–93%) of targets was intron-containing protein-coding genes with only a small proportion of noncoding or other RNAs (Supplemental Fig. S3D,E).

**Figure 3.**
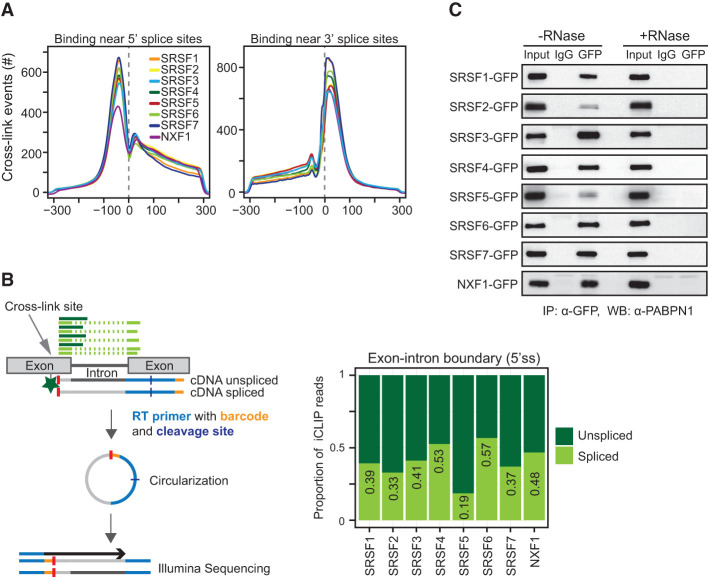
SR proteins and NXF1 bind to mRNA after splicing. (*A*) Number of cross-link events of individual SR proteins mapping to a window of ±300 nucleotides (nt) around the 5′ splice site (*left*) and 3′ splice site (*right*). Each line represents a different SR protein or NXF1. (*B*, *left* panel) Scheme of a two-exon gene with iCLIP reads mapping to the 5′ splice site (5′ss). The cross-link site is indicated with a gray arrow, and the remaining peptide is indicated with a green star. Reads that continue from one exon into the neighboring intron (dark green) were counted as unspliced, and reads that span two neighboring exons (light green) were counted as spliced. (*Right* panel) Plot showing the proportions of spliced and unspliced iCLIP reads mapping near 5′ splice sites. (*C*) Semiquantitative co-IPs using identical conditions with and without RNase A treatment. Pull-downs of mRNPs containing individual GFP-tagged SR proteins were probed for the presence of PABPN1.

If SR proteins recruit NXF1 to mature mRNAs after completion of splicing and dephosphorylation in vivo, then SR proteins should stay bound to spliced mRNA and be detectable in mature mRNPs. We found that SR proteins and NXF1 bind similarly to 5′ and 3′ splice sites (Fig. 3A), suggesting that our iCLIP reads could be used to quantify the splicing status of mRNA targets in vivo. To determine the proportion of cross-linking to spliced and unspliced junctions, iCLIP reads that uniquely mapped to 5′ splice sites of protein-coding genes were compiled and counted (Fig. 3B, left panel). Reads continuing from the exon into the intron (exon–intron) were counted as unspliced, while reads spanning across the junction into the downstream exons (exon–exon) were counted as spliced. This analysis revealed that all SR proteins and NXF1 cross-link to a substantial proportion of spliced junctions (Fig. 3B, right panel), indicating that they maintain direct contacts with mRNA after splicing. This is consistent with the presence of SR proteins in mature mRNPs containing PABPN1 (Fig. 3C). We conclude that a large fraction of SR protein-binding events reflect continuing interactions with mature spliced mRNAs, further supporting potential roles as splicing-sensitive nuclear export adaptors.

### SR proteins and NXF1 cobind within exons

**Figure 4.**
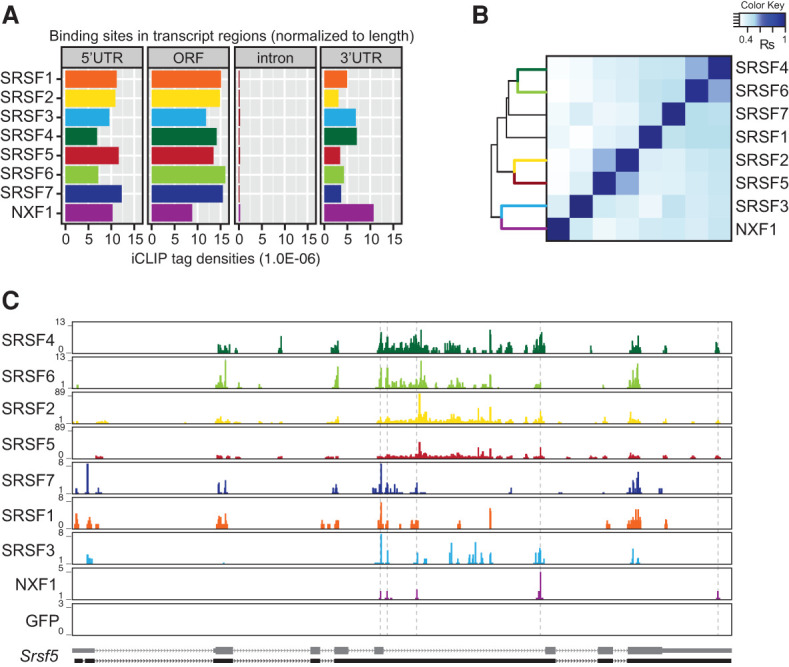
SR proteins and NXF1 cobind within exons. (*A*) Number of significant cross-link events of SR proteins and NXF1 (FDR < 0.05) in different transcript regions normalized to feature length. (*B*) Hierarchical clustering using distance correlation and Spearman rank correlation of exon cobinding between SR proteins and NXF1 using normalized FDR iCLIP tag counts. (*C*) Browser shots showing binding profiles for GFP-tagged SRSF1–7, NXF1, and GFP-NLS on the *Srsf5* gene. SR proteins of the same group share similar binding patterns. *Srsf5* was not detected as export target of any SR protein.

As mRNA export adaptors that recruit NXF1 to mRNA targets, SR proteins and NXF1 are expected to bind mRNA at nearby sites. SR proteins and NXF1 bind predominantly to exon sequences (Fig. 4A). However, NXF1-GFP also binds massively within its own intron 10 (Supplemental Fig. S4A), in which a cytoplasmic transport element (CTE)-like element allows efficient export of this intron-containing *Nxf1* transcript encoding a truncated protein (Li et al. 2006). Indeed, NXF1-GFP also down-regulated expression of endogenous NXF1 protein (Supplemental Fig. S4A), suggesting that NXF1-GFP is functional in our cell line. Whereas SR proteins bound mainly within ORFs, followed by 5′ and 3′ untranslated regions (UTRs), NXF1 bound similarly in 5′ UTRs, ORFs, and 3′ UTRs (Fig. 4A). The high binding density in 3′ UTRs was surprising given that most known export adaptors recruit NXF1 to the 5′ end of pre-mRNAs via the CBC and TREX (Cheng et al. 2006; Nojima et al. 2007; Hautbergue et al. 2009; Katahira et al. 2009). This raised the possibility that SR proteins may recruit NXF1 to the mRNA body and/or 3′ ends.

We investigated whether SR proteins and NXF1 bind to common exons using a normalized number of significant cross-link sites (Supplemental Table S3; Supplemental Fig. S4B). SR protein family members often bound to exons occupied by other SR proteins (Supplemental Fig. S4C). Therefore, we calculated the percentage of “cobound” exons (i.e., exons bound by two or more SR proteins) compared with all bound exons for each SR protein (Supplemental Fig. S4D). Hierarchical clustering revealed that exon binding is highly correlated for SRSF4 and SRSF6 as well as SRSF2 and SRSF5 (Supplemental Fig. S4E). In addition, these SR protein groups displayed similar in vivo binding motifs, as did SRSF1 and SRSF7 (Supplemental Fig. S4E), suggesting that SR protein pairs may compete or cooperate in binding to the same mRNAs. In contrast, SRSF3 displayed the most unique RNA-binding specificity. Its in vivo binding motifs consist of a core CNUC sequence, confirming previous studies (Änkö et al. 2012), and its exon-binding pattern clustered least well with other SR proteins (Supplemental Fig. S4E). Upon inclusion of NXF1 iCLIP data in the clustering analysis, SRSF3 showed the best correlation with NXF1 (Fig. 4B). All other SR proteins were more distant and grouped according to their RNA-binding preferences. These pairwise binding relationships are illustrated for the *Srsf5* gene, on which the grouped SR proteins as well as SRSF3 and NXF1 display similar binding profiles (Fig. 4C).

### NXF1 and SRSF3 bind adjacent sites at the 3′ end of mRNAs

**Figure 5.**
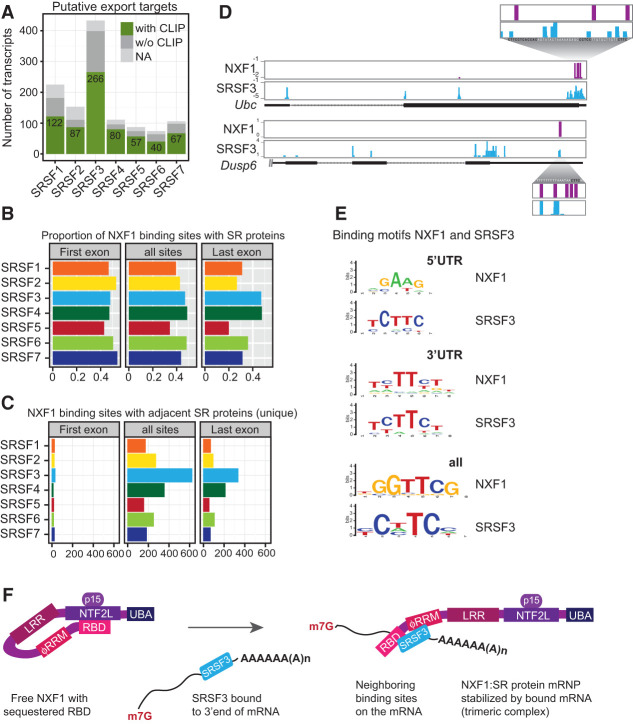
SRSF3 recruits NXF1 to adjacent sites in 3′ UTRs. (*A*) Numbers of export target candidates with (green) or without (dark gray) significant cross-link sites for their cognate SR proteins. (NA) Transcript ID discrepancies in mm9 and mm10 assemblies. (*B*) The occurrence of significant SR protein cross-link sites (*n* > 3) in a small window of 60 nt (±30 nt) around the center of NXF1-binding sites was counted separately in first exons and last exons and over all transcript regions. The ratio of cobinding was calculated with respect to the total number of NXF1-binding sites. (*C*) Number of NXF1-binding sites where only one SR protein cobinds (significant cross-link sites, greater than three). (*D*) Examples of SRSF3 export targets with NXF1- and SRSF3-binding sites in close proximity within last exons, including a blow-up with nucleotide resolution. SRSF3-binding motifs are indicated in black. (*E*) Comparison of in vivo binding motifs (enriched 8-mers) of SRSF3 and NXF1 derived from all transcript regions and separately from 5′ UTRs or 3′ UTRs. (*F*) Working model for NXF1 recruitment and formation of a trimeric complex with bound mRNAs for export based on NXF1 remodeling by TREX (Viphakone et al. 2012). SRSF3 (bound to the 3′ end of mRNAs) binds to NXF1, opens the loop, and unlocks the sequestered RBD, thus enabling NXF1 to bind to adjacent binding sites on the same mRNA. (m7G) Methylguanosine cap; (p15) NXF1 cofactor; (LRR) leucine-rich domain; (NTF2L) nuclear transport factor 2 (NTF2)-like domain; (UBA) ubiquitin-associated domain.

Integration of the iCLIP data with the identified mRNAs dependent on SR proteins for export (see Fig. 1) revealed that a large proportion of mRNA export targets was directly bound by the corresponding SR protein (Fig. 5A). This is consistent with the hypothesis that mRNA binding by SR proteins promotes NXF1 recruitment, as demonstrated for SRSF3 and SRSF7 (see Fig. 2). Visual inspection of SRSF3 and NXF1 iCLIP tags in SRSF3 mRNA export targets suggested that NXF1 is bound to fewer regions relative to the more widespread distribution of SRSF3 iCLIP tags; interestingly, cobinding in these examples occurred mostly in last exons (Supplemental Fig. S5A). Indeed, global analysis showed that most mRNAs had only one or two NXF1-binding sites, predominantly found in 5′ UTRs/first exons or 3′ UTRs/last exons (Supplemental Fig. S5B).

If SR proteins recruit NXF1 to mRNA targets, one might expect SR protein-binding sites and NXF1-binding sites to lie adjacent to one another on target mRNAs. To test this, we analyzed the iCLIP data more stringently, quantifying the cobinding of SR protein within a small window (±30 nucleotides [nt]) around NXF1-binding sites. In first exons and all transcript regions, ∼50% of NXF1-bindings sites were equally cobound by SR proteins (Fig. 5B). This finding is also consistent with our proposal that NXF1–SR protein interactions are stabilized by mRNA binding (see above). In contrast, cobinding among NXF1 and SR proteins differed in last exons, where SRSF4 and SRSF3 bound most often in close proximity to NXF1. In line with this, metagene profiling on last exons showed that SRSF3, SRSF4, and NXF1 had similar cross-linking profiles in last exons, distinct from other SR proteins (Supplemental Fig. S5C).

SRSF3 emerged as the most important single SR protein involved in NXF1 recruitment and mRNA export, following on the observation that it had the most export targets detected upon knockdown (see Fig. 1). Strikingly, SRSF3 was frequently detected within the 60-nt window around NXF1-binding sites in the absence of binding by any other SR protein (Fig. 5C). This was true within both genes and last exons. In contrast, other SR proteins binding within the window often occurred in pairs (Supplemental Fig. S5D). Taken together, this indicates that cobinding between SRSF3 and NXF1 occurs preferentially in last exons.

If NXF1 is directed to mRNA via sequence-specific adaptors, then the adaptor should determine NXF1-binding specificity. For example, Figure 5D shows two examples of SRSF3 export targets with overlapping NXF1-binding sites and SRSF3-binding sites in 3′ UTRs adjacent to bona fide SRSF3-binding motifs. These data suggest that SRSF3 may recruit NXF1 preferentially to last exons or 3′ UTRs, as suggested above, and may also determine the NXF1 binding in this region. To test this globally, in vivo NXF1-binding motifs and SR protein-binding motifs were determined within 5′ UTRs (where TREX is expected to dominate NXF1 recruitment) as well as 3′ UTRs and across all transcript regions (Fig. 5E; Supplemental Fig. S5E). The NXF1-binding motif derived from all regions did not resemble any SR protein motif. Remarkably, the NXF1-binding motif in 3′ UTRs strongly resembled the SRSF3-binding motif (Fig. 5E; Supplemental Fig. S5E), consistent with SRSF3's activity in recruiting NXF1 to adjacent mRNA-binding sites (Fig. 5F).

**Figure 6.**
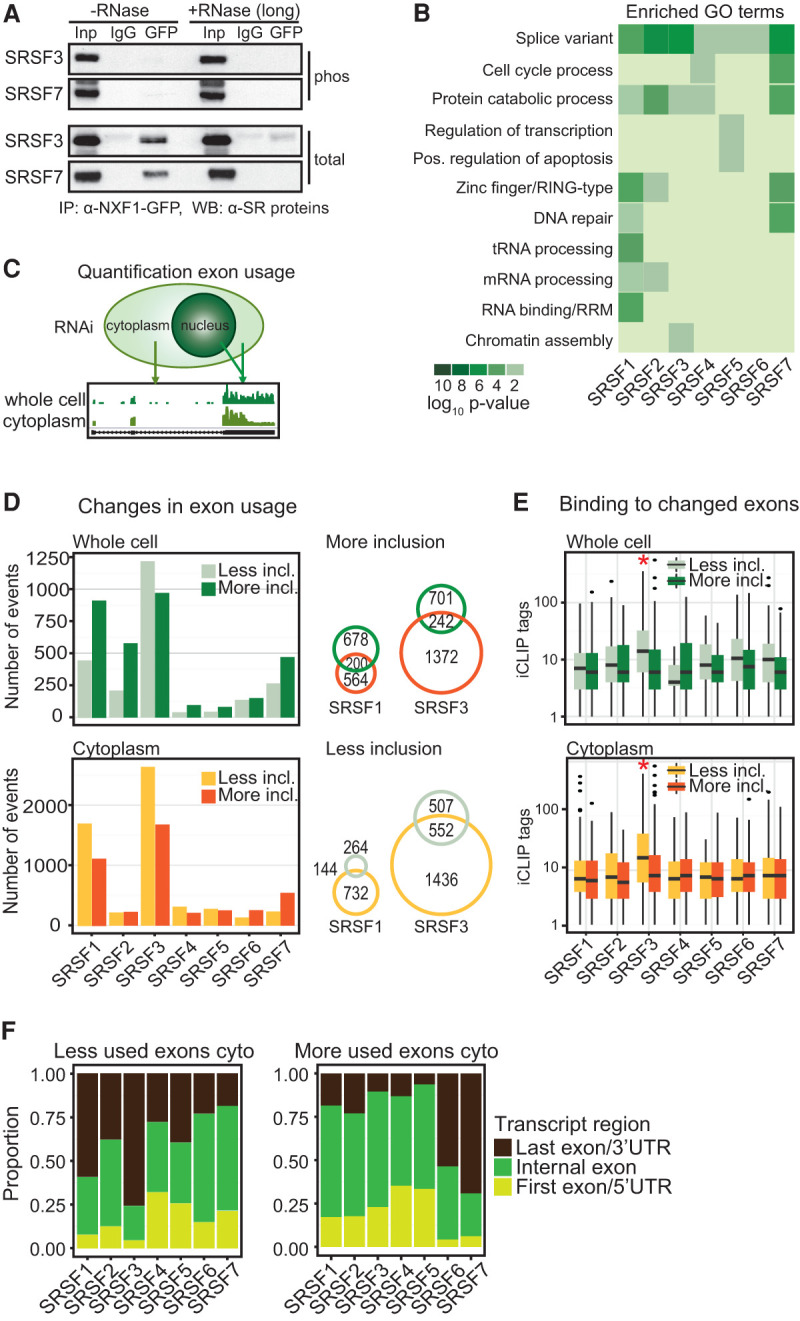
SR proteins link alternative splicing to mRNA export. (*A*) Co-IP of NXF1-GFP with or without RNase A (long) using α-GFP. Phosphorylated SRSF3 and SRSF7 were detected with phosphorylation-dependent mAb104 (top panel) and phosphorylation-independent SR protein-specific (bottom panel) antibodies. (*B*) Gene ontology (GO) term analysis of export targets with significant cross-links of their cognate SR proteins. (*C*) Quantification of exon usage from cytoplasmic and whole-cell RNA-seq after SR protein depletion. (*D*, *left* panel) Changes in exon usage in whole-cell and cytoplasmic RNA samples were calculated using DEX-seq (Anders et al. 2012). *P* < 0.01. Numbers of changed exons after SR protein knockdown were separated in less inclusion and more inclusion. (*Right* panel) Venn diagrams showing coregulated splicing events in total and cytoplasmic samples after SRSF1 and SRSF3 depletion. (*E*) Number of significant cross-link events in changed exons in whole-cell and cytoplasmic RNA. (*F*) Proportion of exons that changed after SR protein knockdowns separated into first exons (light green), internal exons (dark green), and last exons (dark brown).

Our model presupposes that SR proteins recruit NXF1 after splicing, during which SR proteins are dephosphorylated (Huang and Steitz 2005). In agreement with this, SR proteins associated with NXF1 in cells were shown to be hypophosphorylated (Fig. 6A). This is consistent with our demonstration that GFP-tagged SR proteins bind NXF1 (see Fig. 2) and remain bound to spliced transcripts (see Fig. 3). Taken together, our data support a model in which SR proteins are dephosphorylated during splicing, bind NXF1 through protein–protein interactions, induce the RNA-binding-competent conformation of NXF1, and thereby specify NXF1-binding sites adjacent to the SR protein (Fig. 5F).

### SR proteins link alternative splicing to mRNA export

If SR proteins recruit NXF1 after splicing, we would expect mRNA export targets to be functionally distinct or represent different isoforms (Änkö et al. 2010). Indeed, gene ontology (GO) term analysis of the mRNA export targets revealed enrichment of distinct biological processes and the term “splice variant” (Fig. 6B), further suggesting a link between regulated splicing and mRNA export. To test whether SR protein-mediated alternative splicing affects the cytoplasmic abundance of mRNA isoforms, we compared splicing events in whole-cell and cytoplasmic samples and quantified changes in exon usage using DEX-seq (FDR < 0.1) (Fig. 6C,D; Anders et al. 2012). SRSF3 knockdown caused the highest number of splicing changes (Fig. 6D). Of 1988 splicing events altered by SRSF3 knockdown, 1436 were significantly less included in cytoplasmic mRNAs without concomitant changes in whole-cell mRNAs (Fig. 6D). Similar differences (732 cytoplasmic of 876 total changes) were apparent for SRSF1, while depletion of other SR proteins caused fewer alternative splicing changes (Fig. 6D), in line with observed redundancies in RNA binding. Because inclusion of alternative exons is often facilitated by SR protein binding (Han et al. 2011; Erkelenz et al. 2013), we tested for binding of cognate SR proteins within those exons (Fig. 6E). Indeed, most SR proteins displayed a higher number of significant cross-link events in exons that were excluded upon their knockdown, and this difference was most striking for SRSF3 (Fig. 6E, red asterisks).

Because SRSF3 recruits NXF1 to binding sites in last exons (Fig. 5), we hypothesized that SRSF3 might preferentially affect the splicing of last exons. By separating all exons affected by SR protein knockdown into first, internal, or last exons (Fig. 6F), we found that a large proportion of splicing changes occur in first or last exons. Indeed, upon SRSF3 knockdown, 75% of exons with decreased inclusion are last exons. On the other end of the spectrum, SRSF7 depletion caused a much higher number of more included exons, of which 74% are last exons (Fig. 6F). This suggests that SRSF3 and SRSF7 regulate the inclusion of last exons in an opposite direction. It appears that SR proteins in general—and SRSF3 in particular—promote the nuclear export of specific splice isoforms, potentially by influencing the splicing of last exons and subsequent NXF1 recruitment.

### SR proteins link alternative 3′ end formation to mRNA export

**Figure 7.**
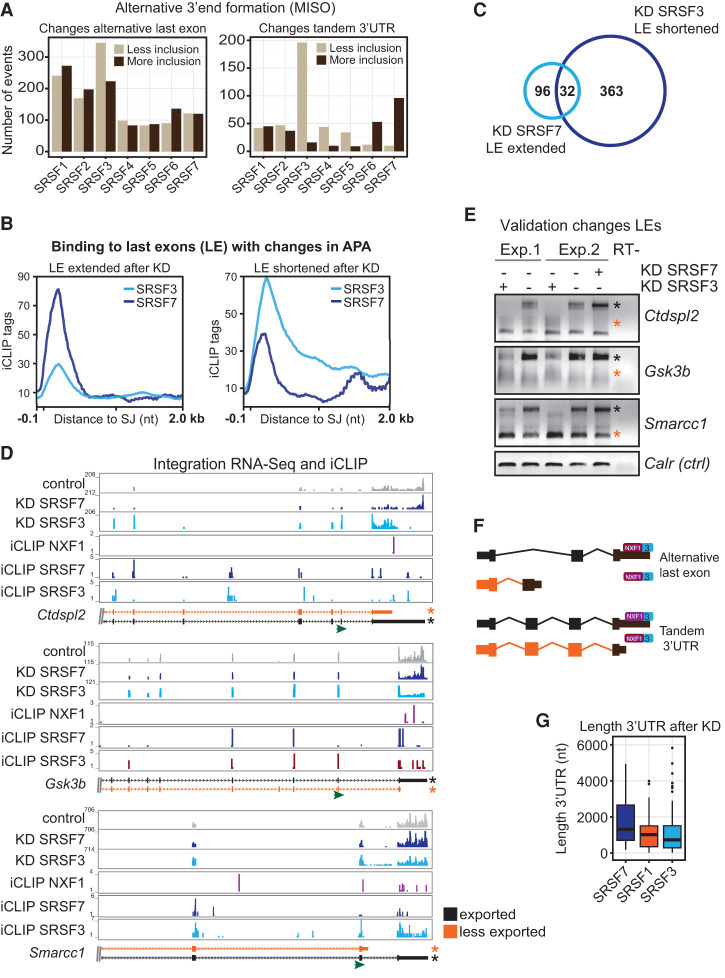
SRSF3 and SRSF7 regulate 3′ UTR length in an antagonistic manner and link alternative 3′ end formation to mRNA export. (*A*) Changes in last exon usage in cytoplasmic samples were mapped to annotated splicing events (ALE and TandemUTR) using MISO (Katz et al. 2010). BF > 5; 20% change. (*B*) Profiles of significant cross-link sites of SRSF3 and SRSF7 in last exons undergoing APA after depletion of the cognate SR protein. (*C*) Venn diagram. Thirty-two genes are regulated in an antagonistic manner by SRSF3 and SRSF7 in APA (cutoff FDR < 0.1). (*D*) Transcripts chosen for validation. Browser shots including affected isoforms (ENSEMBL); RNA-seq read coverage of control RNAi (gray), SRSF3 RNAi (light blue), and SRSF7 RNAi (dark blue); and significant cross-link sites of SRSF3 (light blue), SRSF7 (dark blue), and NXF1 (purple). The transcript isoforms shown are truncated. (Green arrow) Gene-specific forward primer in the penultimate exon for rapid amplification of cDNA 3′ end (3′RACE) RT–PCRs. (*E*) Validation of APA changes after knockdown (KD) of SRSF3 and SRSF7 by 3′RACE RT–PCR. Orange and black asterisks indicate the transcript isoforms shown in *D*. (*F*) Model of how SRSF3 and SRSF7 binding in last exons may affect APA, NXF1 binding, and mRNA export in a splicing-dependent and -independent manner. (*G*) Length distribution of 3′ UTRs from transcripts, which decrease in cytoplasmic abundance after depletion of SRSF7, SRSF1, and SRSF3.

Changes in last exons may stem from alternative terminal exon usage through alternative splicing or from changes in the length of 3′ UTRs in a splicing-independent manner. Both processes alter the site of polyadenylation in the mature mRNA and are therefore referred to as alternative polyadenylation (APA). To test which of these processes is regulated by SR proteins, we mapped changes in last exon usage to known APA annotations (Katz et al. 2010). We found that both types of events were regulated by all SR proteins; notably, the length of tandem 3′ UTRs was most affected in opposite directions upon SRSF3 or SRSF7 knockdown (Fig. 7A). SRSF7 depletion leads to an extension of 3′ UTRs, whereas SRSF3 depletion causes shortening.

In line with this regulation of mRNA 3′ ends, SRSF3 and SRSF7 bind at different positions in affected last exons and 3′ UTRs (Fig. 7B). Last exons extended after SRSF7 depletion showed a strong and sharp peak of SRSF7 binding immediately downstream from last splice junctions and at the beginning of 3′ UTRs, whereas last exons shortened after SRSF3 depletion showed stronger SRSF3 peaks at last splice junctions that extended toward the distal ends of 3′ UTRs (Fig. 7B). Interestingly, 32 genes whose last exons were antagonistically regulated by SRSF3 and SRSF7 were identified (Fig. 7C). We validated antagonistic changes in the usage of alternative terminal exons and/or tandem 3′ UTRs after depletion of SRSF3 and SRSF7 using transcript-specific primers located in the penultimate exons and rapid amplification of cDNA 3′ ends (3′RACE). Following SRSF3 depletion, the abundance of long 3′ UTR isoforms decreased in all cases, while shorter isoforms appeared or increased (Fig. 7D,E, orange asterisks). In contrast, depletion of SRSF7 increased the abundance of isoforms with long 3′ UTRs in most cases (Fig. 7D,E). These data suggest that SRSF3 and SRSF7 and perhaps other SR proteins regulate last exon usage and 3′ UTR length in opposite directions and can even antagonize each other on the same transcripts.

Because NXF1 and SRSF3 show very similar binding patterns in last exons and because NXF1 often binds toward distal ends of 3′ UTRs in SRSF3 export targets (see above), an intriguing possibility is that 3′ UTR shortening or usage of alternative last exons may exclude NXF1 binding from certain alternative mRNAs (Fig. 7F). Reduced binding of NXF1 would decrease the cytoplasmic abundance of these isoforms. In agreement with this possibility, we found that transcripts that are less exported after SRSF3 depletion have shorter 3′ UTRs compared with SRSF1 or SRSF7 targets (Fig. 7G). Investigating SRSF3 export targets in more detail, we found that 265 out of 433 (61%) had SRSF3-binding sites; of those, 171 (64%) were in last exons or 3′ UTRs. Likely due to the rather low expression of export targets in general, we detected NXF1-binding sites in only 126 SRSF3 export targets (29.1%); of those, 80 were found in last exons or 3′ UTRs (63.5%). Strikingly, 40 export targets with NXF1-binding sites underwent APA after SRSF3 depletion; in these cases, polyadenylation cleavage occurs before or within NXF1-binding sites, suggesting that regulated processing can remove RNA sequences that serve as binding sites for NXF1 or NXF1 adaptors (Fig. 7D,E; Supplemental Fig. S6). These data suggest that nuclear export of specific mRNA isoforms is regulated through APA (Fig. 7F,G).

## Discussion

Here we provide evidence that SR proteins play crucial roles as NXF1 adaptors that regulate the export of alternatively processed mRNAs in vivo. Specifically, SR proteins satisfy the following expectations of export adaptors: (1) Individual SR proteins were required for the export of >1000 endogenous mRNAs. (2) SR proteins associated with NXF1 in an RNase A-resistant manner. (3) SRSF3 and SRSF7 promoted NXF1 interactions with mRNA. (4) NXF1 bound to mRNA in close proximity to SR protein-binding sites. SRSF3 and NXF1 often bound together to mRNA downstream regions. (5) Remarkably, NXF1, which lacks sequence-specific binding on its own, exhibited a binding motif similar to that of SRSF3. SRSF3 emerged as the most potent NXF1 adaptor, with the largest number of mRNA export targets of all of the SR proteins. Many of these targets were alternatively spliced and/or polyadenylated, and subsequent analysis revealed that the interplay between SRSF3 and SRSF7 links alternative mRNA processing to mRNA export. These observations support a model in which SR protein binding recruits NXF1 after splicing to promote efficient export of fully processed mRNA. Below, we discuss the basis for these conclusions and expand on related features of SR protein function.

Our iCLIP study revealed that each SR protein binds thousands of transcripts, yet few mRNAs showed changes in cytoplasmic abundance after knockdown. Redundancy and/or cooperation among SR proteins may explain the resistance of mRNA export to depletion of individual SR proteins, as was shown for other export adaptors (Hautbergue et al. 2009; Katahira et al. 2009; Uranishi et al. 2009). Indeed, a severe mRNA export block was achieved upon injection of an antibody against all SR proteins into frog oocytes (Masuyama et al. 2004). Interestingly, our iCLIP data showed that four of the seven SR proteins fell into two paired groups according to their NXF1 interaction and RNA-binding preferences: (1) SRSF4 and SRSF6 and (2) SRSF2 and SRSF5. Depletion of each of these proteins alone led to cytoplasmic reductions in only ∼100 transcripts each, providing evidence for redundancy and compensation. Indeed, depletion of both SRSF4 and SRSF6 is lethal for cells (M Müller-McNicoll, unpubl.), and SR protein pairs exhibited a high degree of cobinding in exons close to NXF1-binding sites. In contrast, SRSF3 bound a unique sequence motif and had the most export targets, suggesting that other SR proteins cannot compensate for loss of SRSF3. Furthermore, SRSF3 often bound at NXF1-binding sites alone and was consistently most abundant in NXF1-containing mRNPs, indicating that, among SR proteins, SRSF3 is the most important NXF1 adaptor.

One of the strongest arguments in favor of SRSF3's role as an NXF1 adaptor is our observation that, although NXF1 itself has no sequence preference, the in vivo binding motif of NXF1 in 3′ UTRs resembles the binding motif of SRSF3. This implicates SRSF3 in the recruitment of NXF1 to specific mRNA 3′ ends. Indeed, our NXF1 iCLIP data revealed thousands of binding sites of NXF1 in 5′ UTRs without specific sequence motifs. However, NXF1-binding sites at the 3′ end of transcripts often overlap with sites where SR proteins bind, particularly SRSF3. Interestingly, we found that most NXF1-binding sites occur at mRNA 5′ and 3′ ends, suggesting that SR proteins and ALYREF independently promote NXF1 recruitment to bulk mRNAs, possibly also in the context of the exon junction complex (Le Hir et al. 2000; Singh et al. 2012). The combination of TREX at 5′ ends and SR proteins in downstream regions seems to provide sufficient adaptor activity to ensure nucleo–cytoplasmic export.

While SRSF3 emerged as a potent NXF1 adaptor, SRSF2 was the least active. For example, overexpression of SRSF3 and SRSF7 promoted NXF1 interaction with mRNA in vivo, while SRSF2 did not. With the exception of SRSF2, most SR proteins shuttle continuously between the nucleus and the cytoplasm (Caceres et al. 1998; Sapra et al. 2009). Consistent with their roles as mRNA export adaptors, SRSF1, SRSF3, and SRSF7 bind directly to NXF1 in vitro (Huang et al. 2003; Lai and Tarn 2004; Hargous et al. 2006; Tintaru et al. 2007). SR proteins enhance NXF1 association with mRNA upon overexpression (see Fig. 2), a hallmark of previously characterized mRNA adaptors (Viphakone et al. 2012). SR proteins only bind NXF1 if they have been dephosphorylated during splicing (Huang et al. 2004; Lai and Tarn 2004; Lin et al. 2005; Sanford et al. 2005); in agreement with these prior findings, we show that SR proteins associated with NXF1 are hypophosphorylated and remain bound to spliced mRNAs.

Our data support mechanistic differences between SR proteins and components of the TREX complex as they relate to NXF1 recruitment and subsequent export. ALYREF and THOC5 “hand over” their bound mRNA cargo to NXF1 after inducing a conformational change that exposes a hidden RBD (Viphakone et al. 2012). This handover is necessary because ALYREF and THOC5 do not shuttle to the cytoplasm and are removed from the mRNP at the nuclear periphery (Kiesler et al. 2002; Golovanov et al. 2006). ALYREF binds RNA nonspecifically and with low affinity via an arginine-rich peptide that overlaps with the NXF1-binding region, making binding mutually exclusive (Hautbergue et al. 2008). In contrast, the RRMs of SR proteins are nonoverlapping with NXF1 interaction regions, suggesting that SR proteins and NXF1 can bind mRNA simultaneously (Hargous et al. 2006; Tintaru et al. 2007).

We favor the hypothesis that SR proteins remain associated with NXF1 and mRNA during export even though previous investigators argued for a handover mechanism by SR proteins to NXF1. First, SR proteins shuttle to the cytoplasm, where they are bound to spliced and polyadenylated mRNAs, are present in translating ribosomes, and regulate translation (Swartz et al. 2007; Sanford et al. 2008; Sato et al. 2008; Änkö et al. 2010; Maslon et al. 2014). Second, SR proteins bind RNA with high affinity and sequence specificity (Cho et al. 2011), unlike the TREX family of adaptors. Third, we show here that SR protein interactions with NXF1 are partially sensitive to RNase A treatment, suggesting stabilization by bound mRNAs. The fact that prolonged RNase treatment disrupts interactions between NXF1 and SR proteins suggests that NXF1 sits on mRNA directly adjacent to its SR protein partner, rendering the RNA linkage relatively less accessible to an enzyme. Fourth, this implied proximity is consistent with our observations of SR protein cobinding with NXF1 in a small (60-nt) window. Therefore, we propose that NXF1, SR proteins, and mRNA form a ternary complex prior to mRNA export, which is transported together to the cytoplasm (Fig. 5F). Although our evidence in support of this model is strongest for SRSF3 and SRSF7, our data suggest that other SR proteins may also perform this role. Thus, SR proteins differ fundamentally from TREX by recruiting NXF1 to 3′ ends and remaining bound at adjacent sites on RNA targets, a prerequisite for their functions in the cytoplasm, such as the translational regulation of specific mRNA isoforms.

Why do cells need so many different export adaptors? Integration of iCLIP and cytoplasmic RNA-seq data revealed a strong link between APA and mRNA export. SR protein depletion affected the cytoplasmic abundance of >1000 endogenous mRNAs, many of which harbored changes in alternative splicing and 3′ end processing. Our data suggest that SRSF3 regulates mRNA export through recruitment of NXF1 to extended 3′ UTRs for the following reasons: (1) SRSF3 binds frequently within last exons compared with other SR proteins. (2) SRSF3 often binds adjacent to NXF1-binding sites in the absence of other SR proteins. (3) SRSF3 and NXF1 display similar binding patterns and in vivo binding motifs in terminal exons. Importantly, SRSF3 depletion leads to a shortening of tandem 3′ UTRs or the inclusion of alternative terminal exons. SRSF3 shows a strong binding peak toward the end of last exons that are shortened after SRSF3 depletion. Finally, SRSF3 export targets often have shortened 3′ UTRs that lack NXF1-binding sites. Remarkably, a function for SRSF3 in alternative terminal exon usage was shown for the *CT/CGRP* pre-mRNA many years ago (Lou et al. 1998), consistent with our present findings.

Finally, we discovered that SRSF3 and SRSF7 regulate 3′ UTR identity and length in an opposite manner and that these choices feed forward to the cytoplasmic abundance of specific isoforms through an export mechanism (Fig. 7F). A function for SRSF7 in APA has not been described thus far. We propose a model in which SRSF3 expression promotes the biogenesis of long 3′ UTR isoforms and regulates their export by preferentially binding at the distal ends of last exons, regulating polyA site selection and recruitment of NXF1. We show that 3′ UTR shortening after SRSF3 depletion can lead to a loss of NXF1-binding sites and reduced export of the shortened 3′ UTR isoforms. In contrast, SRSF7 expression promotes the biogenesis of shorter isoforms, perhaps by recruiting cleavage and polyadenylation factors, and regulates their export. After SRSF7 depletion, NXF1 recruitment fails, and the resulting isoform with a longer 3′ UTR is less exported. Phosphatases present in both splicing and polyadenylation complexes may dephosphorylate SRSF3 and SRSF7 and promote NXF1 recruitment (Shi et al. 2009). Furthermore, recruitment of the export machinery promotes mRNA release from chromatin, whereas incompletely processed transcripts bound by hyperphosphorylated SR proteins are retained at transcription sites (Girard et al. 2012). Taken together, our findings indicate that the expression of alternative mRNA isoforms depends on the orderly action of SR proteins in multiple steps of nuclear pre-mRNA processing, preceding and including mRNA export to the cytoplasm.

## Materials and methods

### Stable BAC cell lines

P19 cells were grown in DMEM GlutaMAX medium (GIBCO) supplemented with 10% heat-inactivated fetal bovine serum (GIBCO), 100 U/mL penicillin, and 100 µg/mL streptomycin (GIBCO) on dishes coated with 0.1% gelatin (Sigma) under humidified 5% CO_2_ at 37°C. GFP-tagged mouse BACs were isolated from *Escherichia coli* DH10 cells using a BAC preparation kit (Macherey-Nagel). P19 cells were transfected with BAC DNA using Effectene (Qiagen), and stable clonal cell lines were obtained after selection with 500 µg/mL Geneticin (GIBCO) and FACS sorting (Supplemental Table S4).

### Knockdown, cell fractionation, and RNA-seq

P19 cells grown until 25% confluency were transfected in six-well plates with 2 µg of custom-made esiRNAs (see the Supplemental Material) using Lipofectamine 2000 (Life Technologies). esiRNAs against GFP were used as a control. Cells were separated into total, nuclear, and cytoplasmic fractions, and RNA was isolated using Trizol (Life Technologies). PolyA^+^ RNA-seq libraries were generated, quantified, and sequenced on an Illumina HiSeq2000 machine obtaining ∼50 million 85-base-pair (bp) or 75-bp single-end reads per sample in two biological replicates (Supplemental Table S1). For validation, RNA was reverse-transcribed using either oligodT primers or RT primers for 3′RACE and SuperScript III (Life Technologies). Quantitative RT–PCR was performed using the SYBR Green kit (Thermo-Fisher) and gene-specific primers. 3′RACE RT–PCR was performed using transcript-specific forward primers located in the penultimate exons and a common reverse primer located in the 3′RACE RT primer.

### OligodT capture

Approximately 5 × 10^7^ P19 cells were irradiated with 0.25 J/cm2 UV light at 254 nm, harvested, and lysed as previously described (Castello et al. 2012). Poly(A)^+^ mRNAs and cross-linked proteins were captured with oligo(dT)_25_ magnetic beads (New England Biolabs). Oligo(dT)_25_ beads were washed with buffers containing decreasing concentrations of LiCl and LiDS, and cross-linked proteins were eluted for 3 min at 55°C , concentrated, and loaded on a 4%–12% NuPAGE gel (Life Technologies). Released NXF1 was analyzed by Western blotting using NXF1-specific antibodies (Santa Cruz Biotechnology).

### iCLIP library preparation

P19 BAC cells were irradiated once with 150 mJ/cm^2^ UV light (254 nm), and iCLIP was performed as described before (Änkö et al. 2012). Protein G Dynabeads coupled with goat anti-GFP antibody (D. Drechsel, Max Planck Institute of Molecular Cell Biology and Genetics [MPI-CBG], Dresden) were used for immunopurification. Cross-linked, immunopurified RNA was digested to lengths of 60–150 nt, reverse-transcribed to generate cDNA libraries, and subjected to high-throughput sequencing on an Illumina HiSeq2000 machine (single-end 75-nt reads).

### Accession numbers

Data are available at Gene Expression Omnibus SuperSeries GSE69734.

## Supplementary Material

Supplemental Material
