# Supplementary material for: SR proteins are NXF1 adaptors that link alternative RNA processing to mRNA export
Source: Genes Dev. 2016 Mar 1;30(5):553–66. doi: 10.1101/gad.276477.115 (PMC4782049; doi:10.1101/gad.276477.115)
Supplement: Supplemental Material [file supp_30_5_553__index.html]

Supplemental Material 

# SR proteins are NXF1 adaptors that link alternative RNA processing to mRNA export

## Supplemental Material

**Files in this Data Supplement:**

- Supp Figure S1.pdf
- Supp Figure S2.pdf
- Supp Figure S3.pdf
- Supp Figure S4.pdf
- Supp Figure S5.pdf
- Supp Figure S6.pdf
- Supp\_Material.pdf
