# Supplementary figures and images for: SR proteins are NXF1 adaptors that link alternative RNA processing to mRNA export
Source: Genes Dev. 2016 Mar 1;30(5):553–66. doi: 10.1101/gad.276477.115 (PMC4782049; doi:10.1101/gad.276477.115)

**A**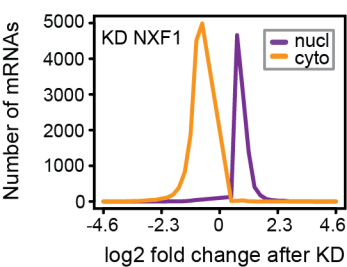**B**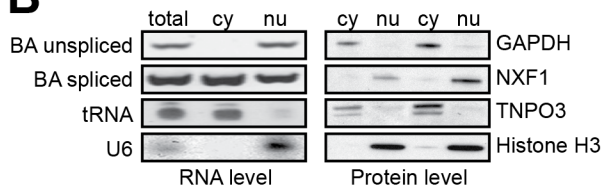**C**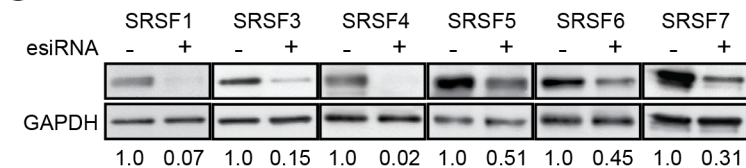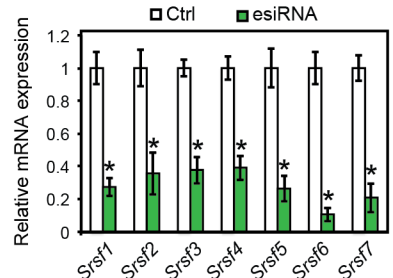**D**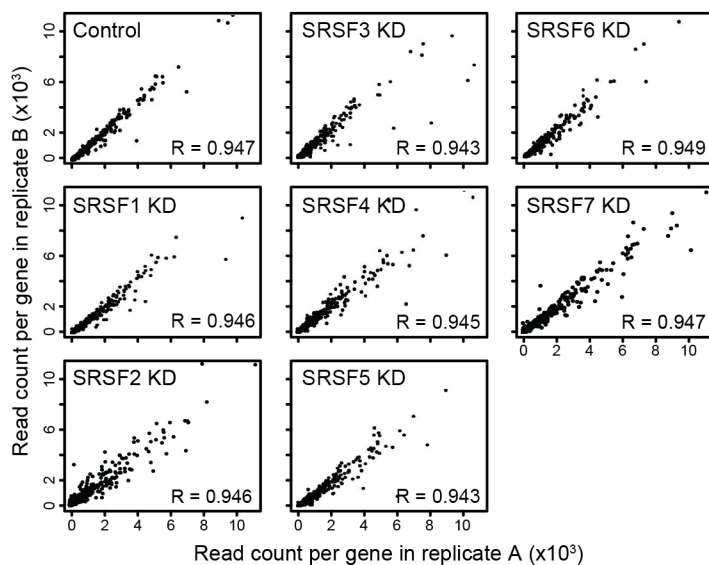**E** Expression changes upon KD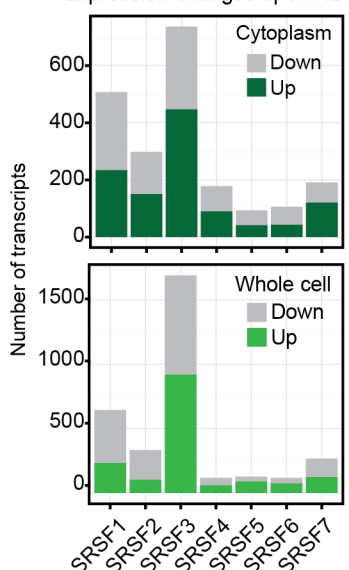**F**

Validation of changes in SRSF3 KD

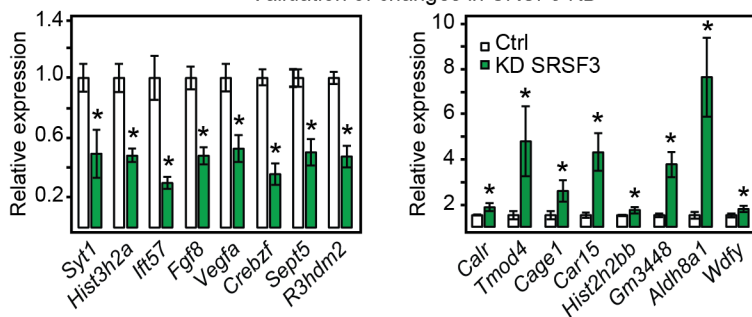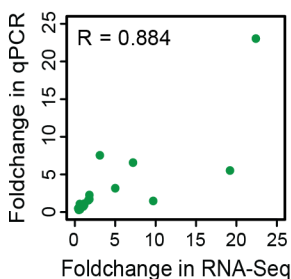**G**

Biotype enrichment

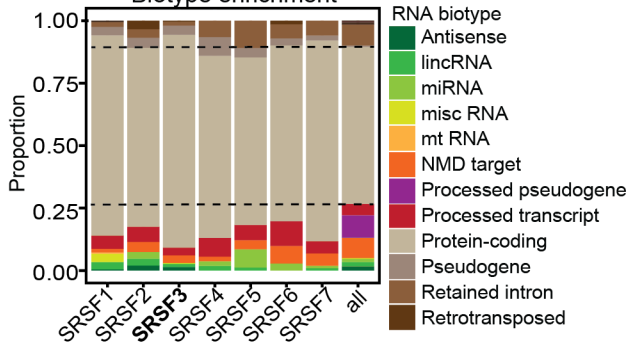**H**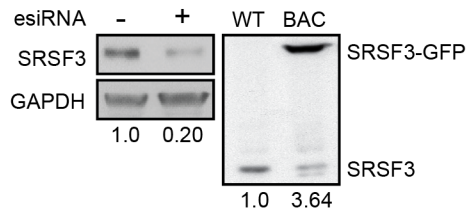

Supplement: Supplemental Material [file supp_30.5.553_SuppFigS1.pdf]

**A**

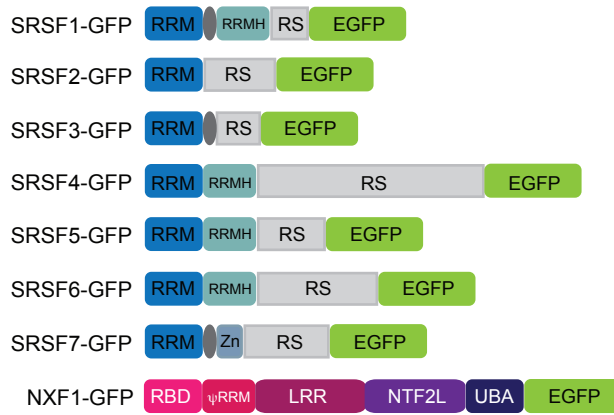

**B**

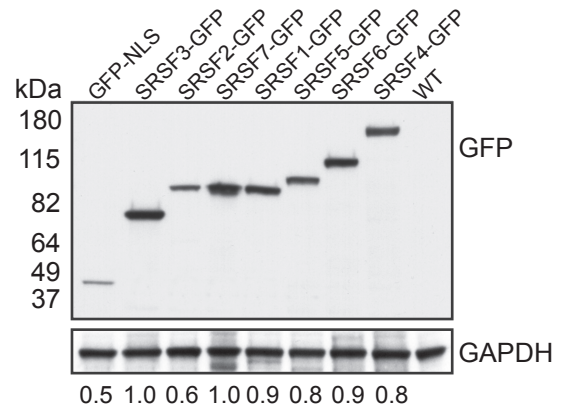

**C**

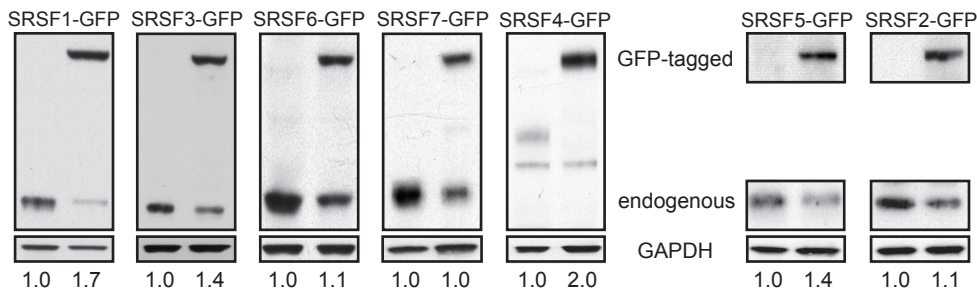

**D**

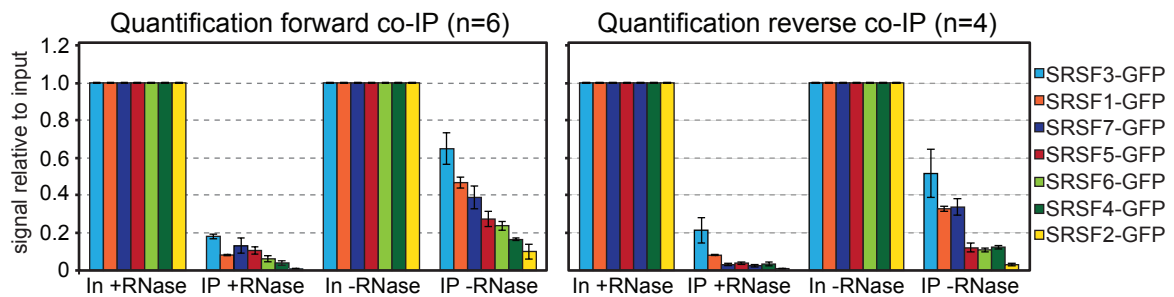

**E**

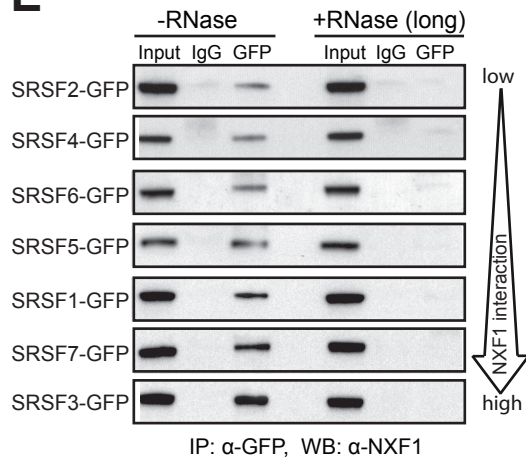

**F**

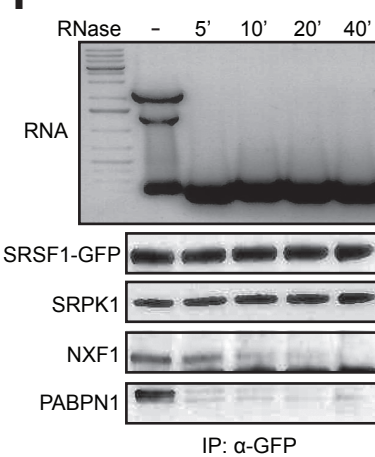

**G**

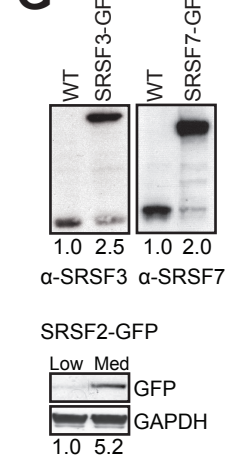

Supplement: Supplemental Material [file supp_30.5.553_SuppFigS2.pdf]

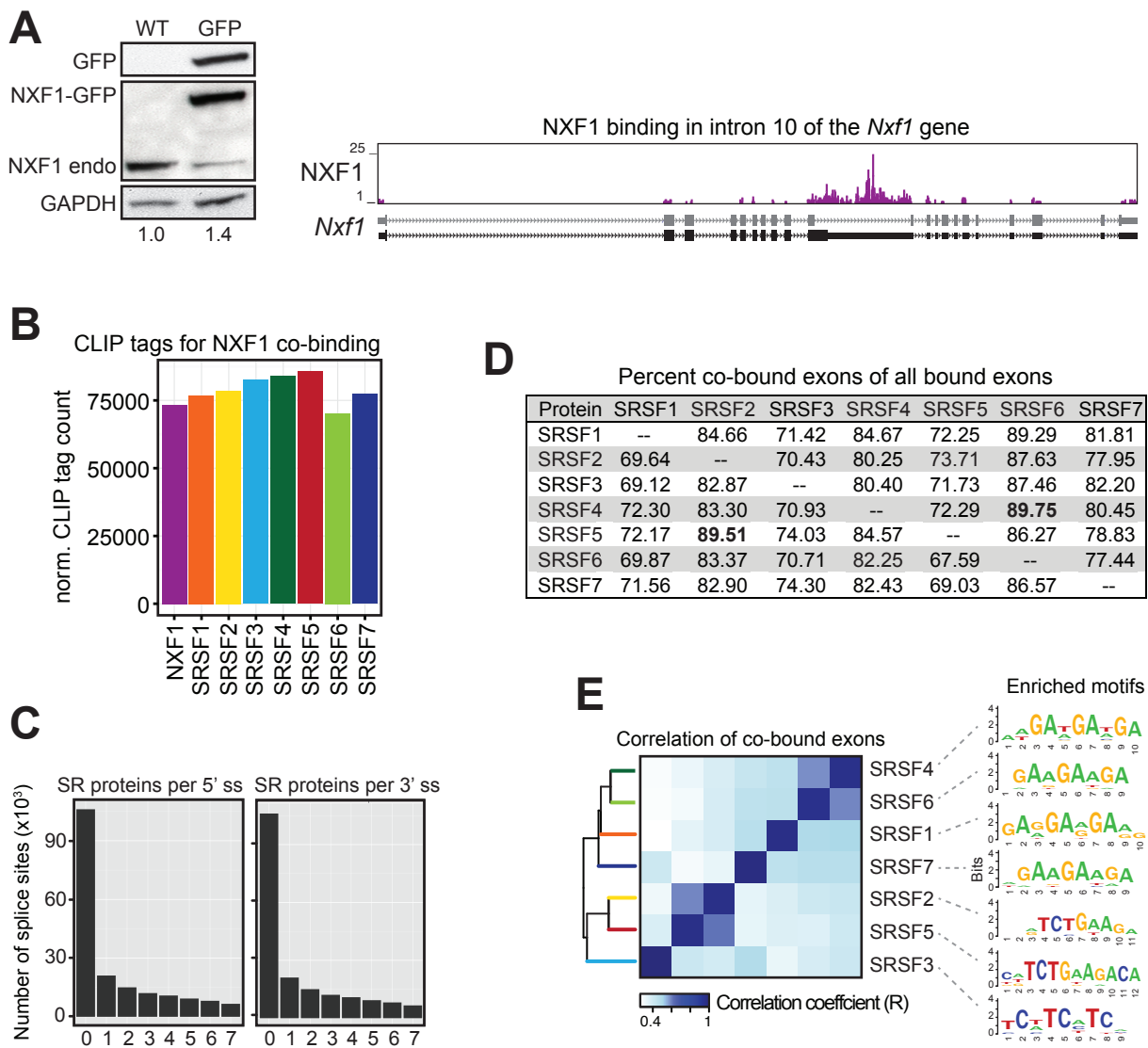

Supplement: Supplemental Material [file supp_30.5.553_SuppFigS4.pdf]

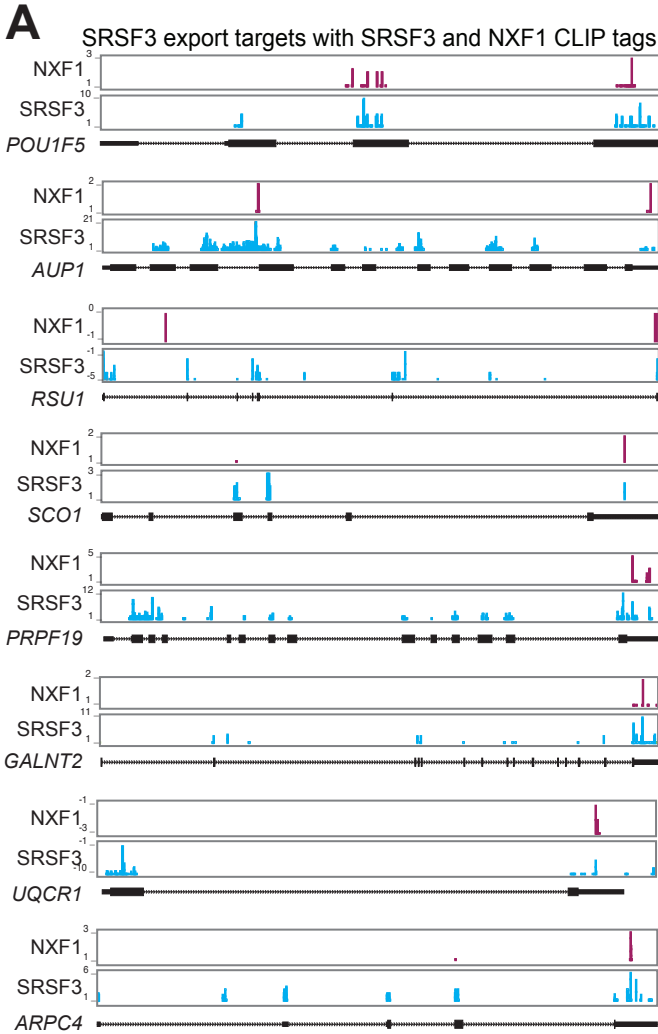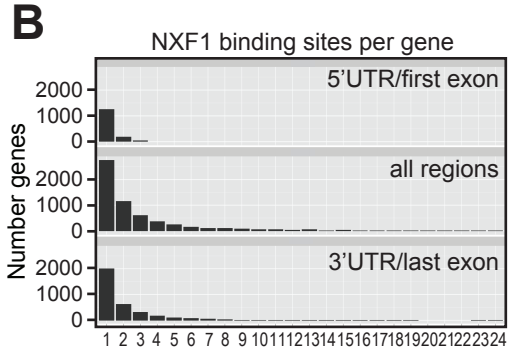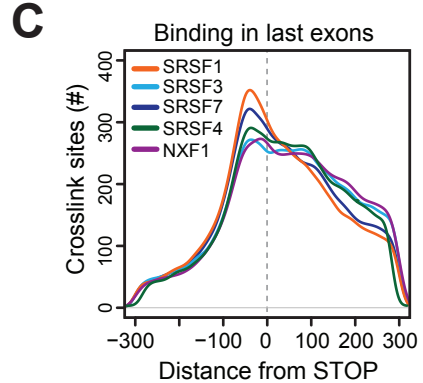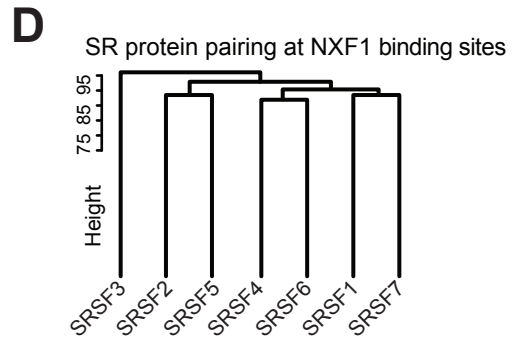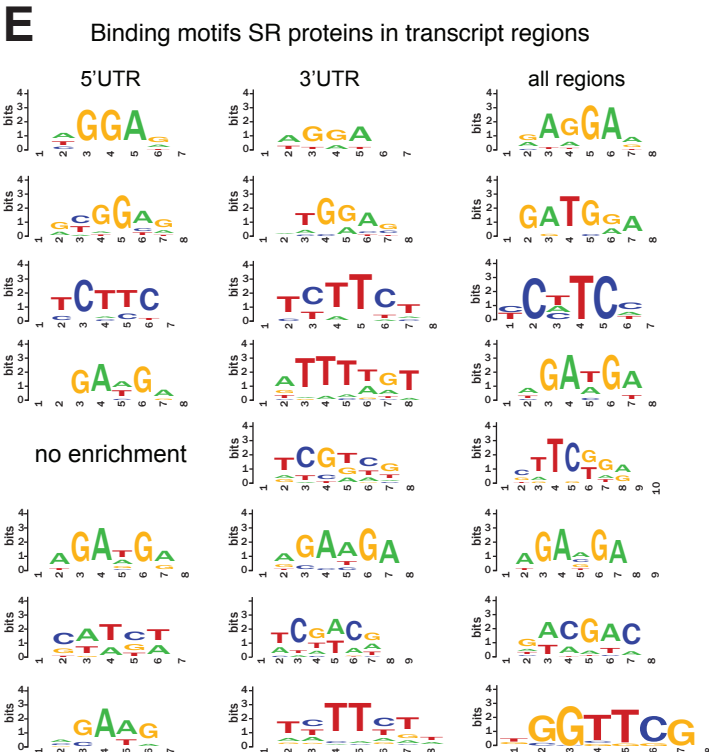

SRSF1

SRSF2

SRSF3

SRSF4

SRSF5

SRSF6

SRSF7

NXF1

Supplement: Supplemental Material [file supp_30.5.553_SuppFigS5.pdf]

**A**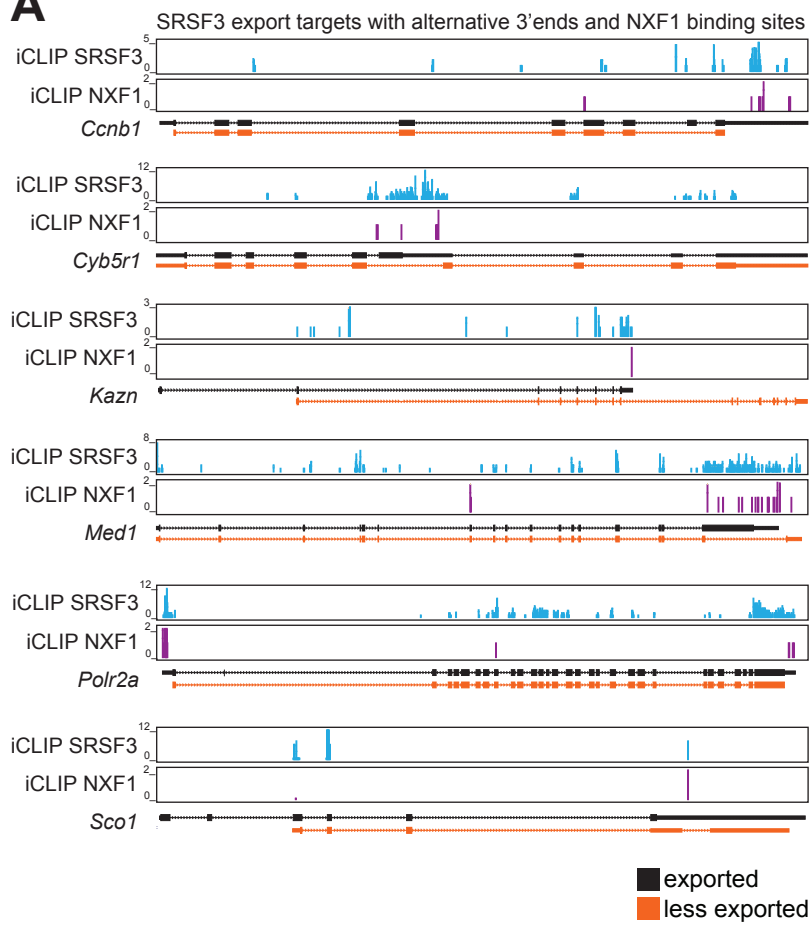

Supplement: Supplemental Material [file supp_30.5.553_SuppFigS6.pdf]
