# Supplementary material for: SR proteins are NXF1 adaptors that link alternative RNA processing to mRNA export
Source: Genes Dev. 2016 Mar 1;30(5):553–66. doi: 10.1101/gad.276477.115 (PMC4782049; doi:10.1101/gad.276477.115)

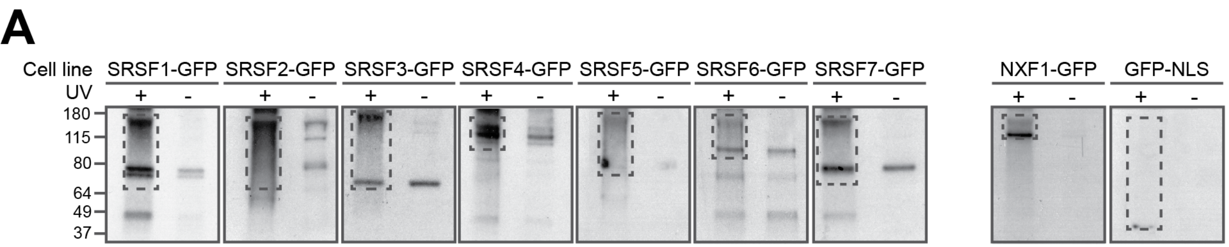

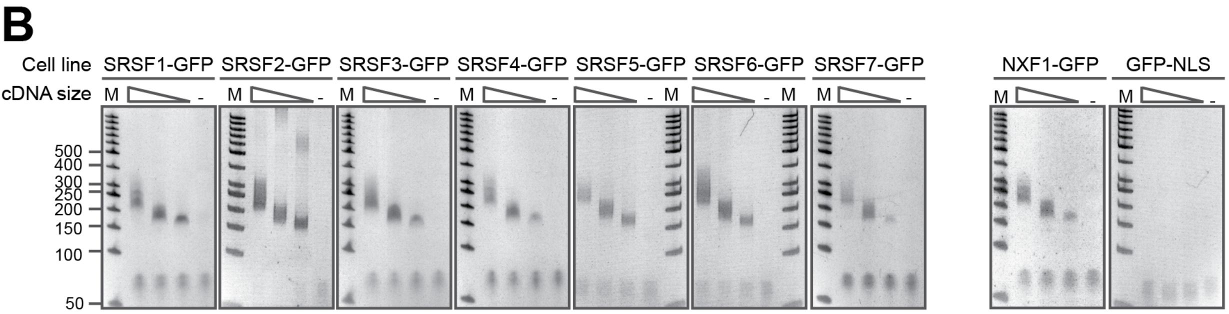

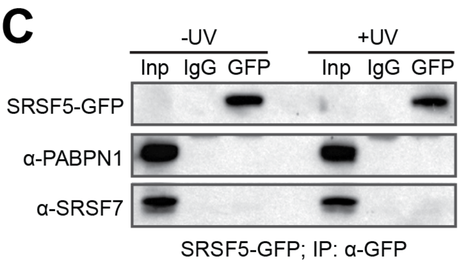

D

Protein	Exons	Genes	PC (%)	Intron-cont.	Intron-less	ncRNAs (%)
SRSF1	42209	8656	89.74	7632	136	10.26
SRSF2	51317	9178	92.31	8382	90	7.69
SRSF3	43613	9142	92.47	8324	130	7.53
SRSF4	49436	8412	90.28	7458	136	9.72
SRSF5	42256	8915	90.56	7978	95	9.44
SRSF6	53939	9199	91.19	8254	135	8.81
SRSF7	48253	8840	91.05	7906	143	8.95
NXF1	48253	5781	85.19	4814	111	14.81

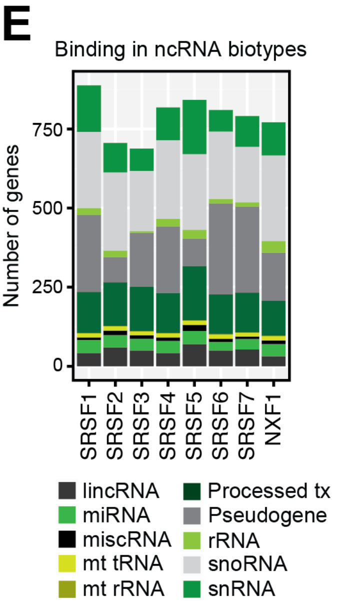

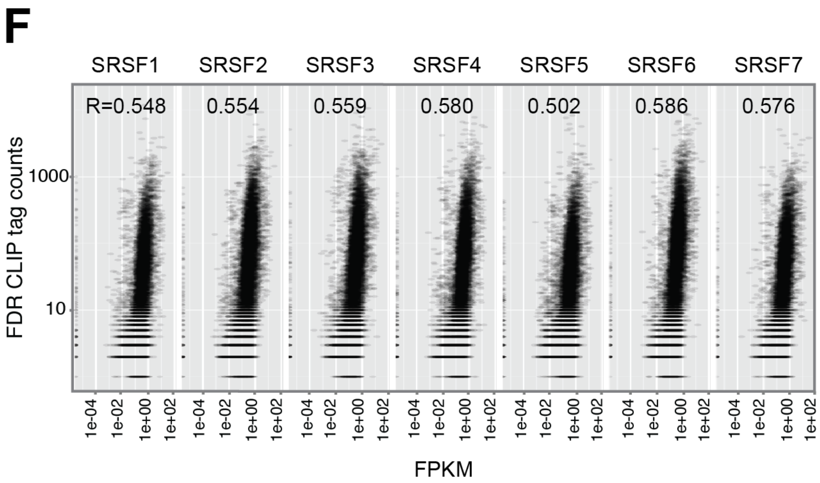

Supplement: Supplemental Material [file supp_30.5.553_SuppFigS3.pdf]
