# Supplementary material for: SR proteins are NXF1 adaptors that link alternative RNA processing to mRNA export
Source: Genes Dev. 2016 Mar 1;30(5):553–66. doi: 10.1101/gad.276477.115 (PMC4782049; doi:10.1101/gad.276477.115)
Supplement: Supplemental Material [file supp_30.5.553_Supp_Material.pdf]

Supplemental material

Supplemental methods

Semi-quantitative co-immunoprecipitation

4x 10^7 cells were lysed in NET-2 buffer supplemented with 1x complete Protease Inhibitor Cocktail (Roche) and 10 mM β-Glycerophosphate (Fluka BioChemica) on ice for 10 minutes. Lysates were sonicated for 30 seconds at 30% amplitude (Branson W-450 D) and cleared by centrifugation. Cell lysates were exposed to 100 μg/ml RNase A or left without and were incubated for 20 minutes at 25˚C or 30 minutes at 37°C (prolonged treatment). For time point experiments, lysates were treated for 5, 10, 20 and 40 minutes at 25˚C. 0.2% of lysate served as input. Ten μg of either mouse-IgG (Sigma), mouse-α-GFP (Roche), goat-IgG (Sigma), goat-α-GFP (D. Drechsel) or goat-α-NXF1 (Santa Cruz) was pre-incubated with gamma-bind sepharose beads (GE Healthcare) for 1 hour at 4°C and then mixed with equal amounts of untreated or RNase A-treated lysates for 1.5 hours at 4°C. Beads were washed, cobound proteins eluted with 2x Laemmli buffer and identified by Western blotting. Quantification was done using FIJI. First, the relative expression of GFP-tagged SR proteins was quantified (as shown in Supplemental Fig. S2C), then the amount of bait that was pulled down was quantified as a loading control. These values were taken into account when normalizing WB signals to the

input. 0.2% of the lysate was used as input in all experiments. The anti-GFP antibody works very efficiently and reproducibly and depletes the lysate almost completely.

Analysis of iCLIP data

Adapters and barcodes were removed from all reads before mapping to the mouse mm9 genome assembly (Ensembl v59 annotation) using Bowtie version 0.10.1. Uniquely mapping reads were kept to determine statistical significant cross-link sites. For this, iCLIP positions were randomized within co-transcribed regions and statistically significant binding sites (false discovery rate [FDR] < 0.05) were calculated using normalized numbers of input cross-link events as previously described (Yeo et al. 2009; König et al. 2010; Wang et al. 2010). To obtain comparable numbers of significant binding sites for all SR proteins, replicates were either pooled (SRSF1, SRSF2, SRSF4, SRSF5, SRSF7) or one replicate was chosen for further analysis, when correlation between replicates was greater than 0.97 (SRSF3, SRSF6).

For exon cobinding, all exons were compiled and the number of significant cross-link events for each SR protein was counted in these features. Exons without cross-link events were removed, the percentage of cobound exons compared to all bound exons was calculated for each SR protein, and hierarchical clustering using distance correlation was performed. Because NXF1 showed ~100 times less binding sites, significant cross-link events of SR proteins were

downscaled and normalized to obtain comparable numbers to analyze NXF1 and SR proteins for exon cobinding.

For motif searching, a z-score analysis for enriched k-mers was performed as described (Wang et al. 2010). Sequences surrounding each significant FDR binding site were extended in both directions by 30 nucleotides. A short window was preferred to exclude the conserved AG nucleotides at 3′ splice sites. Only one occurrence of a k-mer within the evaluated interval (-30, -5), (5, 30) relative to each binding site was counted and weighted by 1.0. Reference data were generated 100 times by random shuffling of iCLIP tag positions within corresponding genome segments (within the same genes) and a z-score was calculated relative to the randomized genomic positions. The top 15 k-mers were used to calculate the in vivo binding consensus motif. Enriched pentamers and octamers were analyzed, and 10 nt around the binding sites were excluded from the analysis to avoid any bias due to differences in cross-linking efficiency of different nucleotides.

To quantify the ratio of spliced and unspliced RNAs bound to individual SR proteins, the genomic regions of all annotated 5′ splice sites in the mouse genome were compiled, and pooled iCLIP reads were mapped to these sites (window +/-50 nt). Only reads that have at least 3 nt overhang across exon borders were considered. Reads that continue from one exon into the

neighboring intron were counted as unspliced, and reads that span two neighboring exons as spliced. 5′ splice sites were chosen because SR proteins bind close to 5′ splice sites and the iCLIP reads start at the cross-link sites owing to the library preparation strategy.

For quantification of NXF1 cobinding, all NXF1 binding clusters (FDR < 0.05) were compiled, divided according to location in 5′UTR/first exon, all regions (including CDS) or 3′UTR/last exon and the co-occurrence of significant cross-link events for individual SR proteins was counted in a window of 60 nucleotides (-30 to +29 nt) around the center of the NXF1 binding site.

Generation of custom esiRNAs

To produce esiRNAs a suitable region of ~400 bp was chosen for each gene using the DEQOR2 suite (Surendranath et al. 2013). Forward and reverse primers were designed each containing the T7 promoter sequence (5′-CGTAATACGACTCACTATAGGG) at the 5' end. Amplified esiRNA target sites were in vitro transcribed (Megascript T7 kit, Ambion) to produce double-stranded RNA (dsRNA), which was subsequently digested to small fragments of 18-25 bp length by RNase III (gift from D. Drechsel, (Theis and Buchholz 2010)) and purified.

Quantification of expression and splicing changes

Microarrays were analyzed using the Gene Spring Software (Agilent). RNA-Seq reads were trimmed and mapped to the mouse genome assembly version mm10 using TopHat v2.0.9 coupled with Bowtie v2.1.0. Reads per kilobase per million mapped reads (RPKM) values were quantified using Cufflinks v2.1.1. (Ensembl v73; gtf=all transcripts). To extract putative export targets, low abundance (RPKM>0.5) or predominantly nuclear transcripts (RPKM ratio of whole cell/cytoplasm > 3) were removed from the cytoplasmic data set, and significant changes ($P < 0.05$) that occur to the same extent in both compartments were filtered out to exclude effects of transcription, splicing or stability on mRNA expression. Transcripts with at least 1.5-fold net decrease in cytoplasmic abundance were considered putative export targets.

Changes in exon usage were calculated using DEXSeq (Anders et al. 2012). For this, mm10 gene annotations (Ensembl v66) were "flattened" and exons split into non-overlapping counting bins. Mapped reads that overlapped with each bin were counted using htseq-count and merged for technical replicates. Counts were used to calculate differential expression of exons using a generalized linear model (FDR < 0.1). All splicing events changed after SR protein knockdown were mapped back to true annotated exons (Ensembl v66) and divided into first exons, internal exons, and last exons based on their exon ranks.

Changes on annotated alternative last exons (ALE) and tandem UTRs were quantified using MISO applying Bayesian inference to estimate exon expression and differential use between conditions (Katz et al. 2010). For this, reads were trimmed to the same length (75 bp) and mapped to mm9 using TopHat (v2.0.10) with the option -g1. The MISO pipeline was followed to estimate exon expression and differential usage with splice annotation provided by the MISO website (https://miso.readthedocs.org/en/fastmiso/annotation.html). MISO does not handle replicates, therefore mapped reads were pooled and Percent Spliced-In (PSI) values and confidence intervals were calculated and compared between knockdown samples and controls. Only differential splicing events that passed a cut-off filter (delta PSI>0.20; Bayes factor>5) were retained for further analysis.

Supplementary figure legends

Supplemental Figure S1 (related to Figure 1). RNA-seq – controls and validation. A) Left panel: Expression changes after NXF1 depletion were quantified separately in cytoplasmic and nuclear samples using whole-genome mouse microarrays in triplicate (Agilent). Numbers of transcripts with significant changes (P < 0.05) were plotted against their corresponding log2 fold change values compared to mock-transfected cells. As control served esiRNAs directed against GFP. **Right panel:** Venn diagram displaying the overlap of transcripts whose levels went down in the cytoplasm and concomitantly up in the nucleus. **Lower panel:** NXF1 was knocked down in P19 wild-type cells using custom-made esiRNAs (Theis and Buchholz 2010). Knockdown efficiency was monitored using a specific antibody for NXF1. To avoid cell death, esiRNA transfections were kept short (24 h). **B) Left panel:** Fractionation efficiency into cytoplasmic (cy) and nuclear (nu) fractions was monitored at the RNA level by RT-PCR using primers specific for spliced or unspliced beta-actin (BA), on stained UREA gels to visualize tRNAs or by Northern blotting using a probe specific for U6 snRNA. **Right panel:** Fractionation efficiency was monitored at the protein level by Western blotting (WB) using antibodies specific for nuclear proteins (NXF1, Histone H3) and cytoplasmic proteins (GAPDH, TNPO3). **C)** SR proteins were

knocked down using esiRNAs. **Left panel:** Knockdown efficiency controlled at the protein level using SR protein-specific antibodies or mAb104. **Right panel:** Knockdown efficiency monitored at the RNA level by RT-qPCR. (*) $P < 0.05$ **D)** Each RNA-Seq experiment was performed in quadruplicate (two biological and two technical replicates). The technical replicates were pooled and the correlation between the biological replicates (replicate A and B) was analyzed using Spearman's rank order correlation of RNA expression level (RPKM). The correlation coefficient (R_S) is shown on the plots. **E)** Differential transcript expression after SR protein knockdown was calculated separately in whole-cell and cytoplasmic samples using Cufflinks ($P < 0.05$; fold change > 1.5-fold) (Trapnell et al. 2013). **F)** Validation of differentially expressed mRNAs in whole-cell RNA-Seq samples by RT-qPCR. Eight mRNAs that were down-regulated (left panel) and eight mRNAs that were up-regulated after SRSF3 knockdown (middle panel) were validated by RT-qPCR. Relative fold changes (FC) obtained by qPCR were plotted against relative FCs of RPKM values. Their correlation was determined using Spearman's rank order correlation R_S (right panel). (*) $P < 0.05$. **G)** Transcript biotype enrichment analysis for candidate export targets of all SR proteins compared to all transcripts expressed in the cytoplasmic RNA-Seq data set (all). **H)** Quantification of knockdown and overexpression efficiency of SRSF3 for validation of export targets by WB relative to WT cells.

Supplemental Figure S2 (related to Figure 2) SRSF3 is the most prominent SR protein detectable in NXF1-containing mRNPs.

A) Scheme of the seven GFP-tagged SR proteins and NXF1-GFP used in this study. **B)** Western blot showing that GFP-fusion proteins are expressed at similar levels in P19 cells and have the correct size. **C)** WB on P19 WT cells and GFP-lines using SR protein-specific antibodies or mAb104. Expression of GFP-tagged SR proteins relative to endogenous proteins is quantified. Numbers are shown below the blots. **D)** Quantification of SR protein:NXF1 co-IP relative to input (In) and normalized to bait from 6 (forward) or 4 (reverse) independent experiments performed under identical conditions. **E)** NXF1 interaction with SR proteins is completely abolished after prolonged RNase treatment. P19 cell lysates were incubated without or with RNase A for 20 minutes. GFP-tagged SR proteins were immunoprecipitated using anti-GFP antibodies and probed for interacting NXF1. **F)** Time course of RNase A treatment using SRSF1-GFP expressing cells. GFP-tagged SRSF1 was immunoprecipitated by anti-GFP antibodies and probed for interacting proteins with antibodies specific for NXF1, SRPK1 and PABPN1. An RNA gel from lysates of all time points demonstrates complete RNA degradation. **G)** Quantification of SRSF2, SRSF3 and SRSF7 overexpression in P19 clonal cell lines selected for high GFP expression by FACS using WB relative to WT cells.

Supplemental Figure S3 (related to Figure 3). iCLIP – library preparation, controls and target genes. A) Protein:RNA complexes were immunopurified from P19 cells expressing GFP-tagged SR proteins or NXF1 after *in vivo* UV cross-linking using anti-GFP antibodies and stringent conditions according to (König et al. 2010). Complexes were visualized by autoradiography and areas above the molecular weight (MW) of the respective protein were cut from the membrane to extract the bound RNA (dashed lines). Immunoprecipitated SR proteins become labeled with γ-^{32}P-labeled ATP, likely due to associated SR protein kinases (Sanford et al. 2008; Pandit et al. 2013), providing a size marker for individual SR proteins. Labelled RNAs were not detectable in cells expressing GFP-NLS or in the absence of UV cross-linking. **B)** iCLIP libraries after size fractionation of GFP-tagged SR proteins, NXF1-GFP and controls in P19 cells. **C)** Test for stringency. Co-IPs using the same UV cross-linking and IP conditions as in the iCLIP protocol were used to ensure that other SR proteins were not co-purified. A representative example is shown for SRSF5-GFP and endogenous SRSF7. **D)** The majority of SR protein targets are protein-coding intron-containing genes with few binding in non-coding RNAs. Number of bound exons and target genes for individual SR proteins and NXF1. PC – protein-coding; ncRNAs – non-coding RNAs. **E)** Proportional binding of individual SR proteins in ncRNA biotypes by number of ncRNA genes. **F)** Scatter plots showing the correlation between FDR

iCLIP tag counts and expression of the cognate mRNAs in reads per kilobase per million mapped (RPKMs) (quantified from P19 control whole-cell RNA-Seq data). Spearman correlation coefficients (Rs) are shown on the top of the plots.

Supplemental Figure S4 (related to Figure 4). SR proteins form groups with similar RNA binding preferences and in vivo binding motifs and cobind with NXF1 in exons. A) Left panel: GFP-tagged NXF1 auto-regulates endogenous protein levels, as determined by anti-NXF1 western blotting of extracts from WT P19 and a stable cell line expressing NXF1-GFP from a BAC. **Right panel:** NXF1-GFP binds to a cytoplasmic transport element (CTE) located within a retained intron (intron 10) of its own transcript (black). **B)** Normalized number of significant binding sites (FDR iCLIP tags) included in the comparison of NXF1 and SR protein cobinding. **C)** Cobinding of SR proteins at splice sites. **Left panel:** Number of 5′ splice sites (5′ss) occupied by none up to all seven SR proteins. **Right panel:** Number of 3′ splice sites (3′ss) occupied by none up to all seven SR proteins. **D)** Table showing the proportion (in percent) of cobound exons to total bound exons for all SR proteins. **E) Left panel:** Hierarchical clustering, using distance correlation and Spearman rank correlation of exon cobinding showing SR protein pairs often binding to the same exons. **Right panel:** In vivo binding motifs of individual SR proteins derived through analysis of enriched octamers surrounding significant cross-link sites in all transcript regions. Ten nucleotides

around the cross-link site were excluded to avoid stretches of T due to cross-linking bias (window -30 to -5, +5 to +30).

Supplemental Figure S5 (related to Figure 5). SRSF3 recruits NXF1 to adjacent sites in 3′ UTRs. A) Browser shots showing examples of SRSF3 candidate export targets with adjacent NXF1 (purple) and SRSF3 (light blue) binding sites in last exons. **B)** Number of NXF1 binding sites per gene, separated by first or last exons and within all regions including introns. Significant binding sites were pooled when they were less than 10 nt apart, yielding a total of 22,733 NXF1 binding sites. **C)** Metagene cross-link profiles in last exons aligned at the ORF-3′ UTR boundary for SRSF4, SRSF3, SRSF1, SRSF7 as well as NXF1. **D)** SR protein binding at NXF1 binding sites using normalized FDR iCLIP tags and Spearman's rank correlation, shown as distance dendrogram. SR proteins with similar RNA binding preferences cobind around NXF1 binding sites. **E)** Comparison of in vivo binding motifs (enriched 5-mers) of all SR proteins and NXF1 derived from all transcript regions and separately from 5′ UTRs or 3′ UTRs.

Supplemental Figure S6 (related to Figure 7). 3′ UTR shortening reduces NXF1 binding sites and export of SRSF3 targets. A) Browser shots showing genes for SRSF3 export targets including the exported isoform (black) and the retained isoforms that is altered in its 3′ end after SRSF3 KD (orange) often removing binding sites for NXF1. RNA-Seq read coverage of SRSF3 (light blue) and SRSF7

(dark blue) knockdown and significant cross-link sites of SRSF3 (light blue), SRSF7 (dark blue) and NXF1 (purple) are indicated.

Supplemental Tables

Supplemental Table S1. RNA-Seq mapping statistics

**Control RNA-Seq**	Replicate A whole cell	Replicate B whole cell	Replicate A cyto	Replicate B cyto
Trimmed reads	33,393,631	39,250,362	31,205,382	39,247,879
Mapped reads	32,121,013	37,805,554	30,126,862	38,041,887
Mapping efficiency	96.19%	96.32%	96.54%	96.93%

**KD SRSF1 RNA-Seq**	Replicate A whole cell	Replicate B whole cell	Replicate A cyto	Replicate B cyto
Trimmed reads	37,068,345	36,761,886	39,836,108	40,693,602
Mapped reads	35,586,584	35,290,636	38,431,967	39,416,836
Mapping efficiency	96.00%	96.00%	96.48%	96.86%

**KD SRSF2 RNA-Seq**	Replicate A whole cell	Replicate B whole cell	Replicate A cyto	Replicate B cyto
Trimmed reads	34,331,305	33,652,944	30,117,043	36,652,526
Mapped reads	32,965,222	32,437,491	28,978,828	35,400,345
Mapping efficiency	96.02%	96.39%	96.22%	96.58%

**KD SRSF3 RNA-Seq**	Replicate A whole cell	Replicate B whole cell	Replicate A cyto	Replicate B cyto
Trimmed reads	35,581,818	41,056,388	33,834,104	49,313,290
Mapped reads	34,176,948	39,525,330	32,608,649	47,765,450
Mapping efficiency	96.05%	96.27%	96.38%	96.86%

**KD SRSF4 RNA-Seq**	Replicate A whole cell	Replicate B whole cell	Replicate A cyto	Replicate B cyto
Trimmed reads	42,401,440	35,659,550	31,143,568	41,285,344
Mapped reads	40,861,954	34,363,265	29,876,390	40,062,041
Mapping efficiency	96.37%	96.36%	95.93%	97.04%

**KD SRSF5 RNA-Seq**	Replicate A whole cell	Replicate B whole cell	Replicate A cyto	Replicate B cyto
Trimmed reads	41,435,699	40,397,221	33,759,000	52,366,189
Mapped reads	39,863,538	38,919,279	32,353,893	50,822,689
Mapping efficiency	96.21%	96.34%	95.84%	97.05%

**KD SRSF6 RNA-Seq**	Replicate A whole cell	Replicate B whole cell	Replicate A cyto	Replicate B cyto
Trimmed reads	40,525,703	51,654,866	36,679,511	41,790,476
Mapped reads	39,006,025	49,850,853	35,272,250	40,553,328
Mapping efficiency	96.25%	96.51%	96.16%	97.04%

**KD SRSF7 RNA-Seq**	Replicate A whole cell	Replicate B whole cell	Replicate A cyto	Replicate B cyto
Trimmed reads	37,086,896	48,535,122	38,912,411	47,166,985
Mapped reads	35,660,715	46,791,167	37,480,209	45,480,908
Mapping efficiency	96.15%	96.41%	96.32%	96.43%

Supplemental Table S2. Replicate correlations iCLIP, related to material and methods

	Replicate 1	Replicate 2	Replicate 3	Replicate 4	Replicate 5	Replicate 6
**GFP**	1	0.212533883	0.283253793	0.313229663		
**GFP**	0.212533883	1	0.272165512	0.362179327		
**GFP**	0.283253793	0.272165512	1	0.412062565		
**GFP**	0.313229663	0.362179327	0.412062565	1		
**NXF1**	1	0.943083446	0.922673581			
**NXF1**	0.943083446	1	0.912715636			
**NXF1**	0.922673581	0.912715636	1			
**SRSF1**	1	0.969956428	0.921493953	0.38618817		
**SRSF1**	0.969956428	1	0.911698862	0.39679361		
**SRSF1**	0.921493953	0.911698862	1	0.410681705		
**SRSF1**	0.38618817	0.39679361	0.410681705	1		
**SRSF2**	1	0.86280551	0.738472974	0.878026583	0.863294813	
**SRSF2**	0.86280551	1	0.756894765	0.921044112	0.982787875	
**SRSF2**	0.738472974	0.756894765	1	0.787510626	0.769819009	
**SRSF2**	0.878026583	0.921044112	0.787510626	1	0.933955307	
**SRSF2**	0.863294813	0.982787875	0.769819009	0.933955307	1	
**SRSF3**	1	0.977780296	0.973146266			
**SRSF3**	0.977780296	1	0.985265242			
**SRSF3**	0.973146266	0.985265242	1			
**SRSF4**	1	0.925497795	0.908321947			
**SRSF4**	0.925497795	1	0.950272525			
**SRSF4**	0.908321947	0.950272525	1			
**SRSF5**	1	0.883535987	0.865340399	0.870194955		
**SRSF5**	0.883535987	1	0.933326273	0.893106761		
**SRSF5**	0.865340399	0.933326273	1	0.857472255		
**SRSF5**	0.870194955	0.893106761	0.857472255	1		
**SRSF6**	1	0.973141973	0.978225175			
**SRSF6**	0.973141973	1	0.991024489			
**SRSF6**	0.978225175	0.991024489	1			
**SRSF7**	1	0.756239839	0.75641066	0.825457108	0.655565884	0.766854786
**SRSF7**	0.756239839	1	0.838464021	0.881683644	0.728464862	0.879332427
**SRSF7**	0.75641066	0.838464021	1	0.870139282	0.719300604	0.873300585
**SRSF7**	0.825457108	0.881683644	0.870139282	1	0.761510327	0.946115463
**SRSF7**	0.655565884	0.728464862	0.719300604	0.761510327	1	0.777765558
**SRSF7**	0.766854786	0.879332427	0.873300585	0.946115463	0.777765558	1

Supplemental Table S3. iCLIP mapping statistics, related to material and methods

GFP-NLS iCLIP (Ctrl)	Replicate 1	Replicate 2	Replicate 3	Replicate 4		Combined
Total reads	14,812,822	4,786,440	12,177,392	13,648,468		45,425,122
Cross-link (X-link) events	9,772	6,192	15,835	6,780		38,579
Sign. X-link events (FDR < 0.05)	ND	ND	ND	ND		8,259
Binding sites	ND	ND	ND	ND		2,196
**SRSF1-GFP iCLIP**	Replicate 1	Replicate 2	Replicate 3	Replicate 4		Combined
Total reads	20,856,758	32,637,942	37490868	18,076,695		109,062,263
Cross-link (X-link) events	2,153,384	887,107	289,347	15,623		3,345,461
Sign. X-link events (FDR < 0.05)	ND	ND	ND	ND		1,333,834
Binding sites	ND	ND	ND	ND		125,475
**SRSF2-GFP iCLIP**	Replicate 1	Replicate 2	Replicate 3	Replicate 4	Replicate 5	Combined
Total reads	32,610,168	30,950,360	30,415,725	17,546,058	27,062,766	138,585,077
Cross-link (X-link) events	5,867,819	921,268	5,152,490	92,851	202,860	12,237,288
Sign. X-link events (FDR < 0.05)	ND	ND	ND	ND	ND	2,147,521
Binding sites	ND	ND	ND	ND	ND	190,878
**SRSF3-GFP iCLIP**	Replicate 1	Replicate 2	Replicate 3			Combined
Total reads	23,372,667	24,163,627	30,735,922			78,272,216
Cross-link (X-link) events	1,682,594	6,523,851	6,145,572			14,352,017
Sign. X-link events (FDR < 0.05)	ND	ND	ND			1,949,249
Binding sites	ND	ND	ND			214,307
**SRSF4-GFP iCLIP**	Replicate 1	Replicate 2	Replicate 3			Combined
Total reads	23,308,234	28,101,424	25,091,553			76,501,211
Cross-link (X-link) events	297949	3575481	690483			4,563,913
Sign. X-link events (FDR < 0.05)	ND	ND	ND			1,841,695
Binding sites	ND	ND	ND			172,833
**SRSF5-GFP iCLIP**	Replicate 1	Replicate 2	Replicate 3	Replicate 4		Combined
Total reads	17,792,219	31,709,513	38,940,161	25,605,015		88,441,893
Cross-link (X-link) events	258,727	1,414,674	1,941,760	258,983		3,874,144
Sign. X-link events (FDR < 0.05)	ND	ND	ND	ND		1,684,467
Binding sites	ND	ND	ND	ND		137,413
**SRSF6-GFP iCLIP**	Replicate 1	Replicate 2	Replicate 3			Combined
Total reads	31,619,659	27,661,710	27,667,471			86,948,840
Cross-link (X-link) events	5,631,019	3,199,736	5,741,825			14,572,580
Sign. X-link events (FDR < 0.05)	ND	ND	ND			2,323,023
Binding sites	ND	ND	ND			194,540

(Continued on next page)

Supplemental Table S3. Continued from previous page

SRSF7-GFP iCLIP	Replicate 1	Replicate 2	Replicate 3	Replicate 4	Replicate 5	Combined
Total reads	28,267,353	31,294,225	28,267,353	34,562,260	13,228,191	148,346,453
Cross-link (X-link) events	2,856,178	203,243	132,937	215,439	69,230	3,477,027
Sign. X-link events (FDR < 0.05)	ND	ND	ND	ND	ND	1,449,645
Binding sites	ND	ND	ND	ND	ND	152,146
**NXF1-GFP iCLIP**	Replicate 1	Replicate 2	Replicate 3			Combined
Total reads	18,810,946	17,619,160	14,833,018			51,263,124
Cross-link (X-link) events	1,010,202	539,280	337,415			1,886,897
Sign. X-link events (FDR < 0.05)	ND	ND	ND			569,674
Binding sites	ND	ND	ND			35,741

Supplemental Table S4. Stable P19 cell lines used in this study, related to material and methods

Name	Construct	Selection Marker	Expression
GFP-NLS	Plasmid	G418	Low
SRSF1-GFP	BAC	G418	Medium
SRSF2-GFP, clone 1	BAC	G418	Low
SRSF2-GFP, clone 11	BAC	G418	Medium
SRSF3-GFP, clone 2L	BAC	G418	Medium
SRSF3-GFP, clone 3H	BAC	G418	High
SRSF4-GFP	BAC	G418	Medium
SRSF5-GFP	BAC	G418	Medium
SRSF6-GFP	BAC	G418	Medium
SRSF7-GFP, clone 4	BAC	G418	Medium
SRSF7-GFP, clone 6	BAC	G418	High
NXF1-GFP	BAC	G418	Medium

Supplemental Table Legends:

Supplemental Table S1. RNA-Seq mapping statistics. Reads were trimmed and mapped to mouse genome assembly mm10 using TopHat v2.0.9 coupled with Bowtie v2.1.0.

Supplemental Table S2. Replicate correlations of iCLIP data sets. Replicate correlation was calculated using the Spearman' rank order correlation based on the number of significant cross-links per gene.

Supplemental Table S3. iCLIP mapping statistics. After removal of adapters and barcodes, reads were mapped to the mouse mm9 genome assembly using Bowtie version 0.10.1. Uniquely mapping reads were used for peak calling and to determine statistical significant cross-link sites (false discovery rate [FDR] < 0.05) using randomization of co-transcribed regions and the modified FDR method (Yeo et al. 2009; König et al. 2010; Wang et al. 2010).

Supplemental Table S4. Stable P19 cell lines used in this study. All transgenes contain a localization and affinity purification (LAP) tag (Poser et al. 2008). For most stable cell lines, FACS selection was used to sort for clonal cell lines with low, medium and high GFP expression. Highly expressing clones we used for overexpression experiments, and medium expressing clones were used for iCLIP or

Co-IPs to best possibly match the endogenous level of the respective SR protein. For SRSF2, only medium and low expression levels were obtained.

Supplemental references:

Anders S, Reyes A, Huber W. 2012. Detecting differential usage of exons from RNA-seq data. *Genome Res* **22**: 2008-2017.

Katz Y, Wang ET, Airoldi EM, Burge CB. 2010. Analysis and design of RNA sequencing experiments for identifying isoform regulation. *Nat Methods* **7**: 1009-1015.

König J, Zarnack K, Rot G, Curk T, Kayikci M, Zupan B, Turner DJ, Luscombe NM, Ule J. 2010. iCLIP reveals the function of hnRNP particles in splicing at individual nucleotide resolution. *Nat Struct Mol Biol* **17**: 909-915.

Pandit S, Zhou Y, Shiue L, Coutinho-Mansfield G, Li H, Qiu J, Huang J, Yeo GW, Ares M, Jr., Fu XD. 2013. Genome-wide analysis reveals SR protein cooperation and competition in regulated splicing. *Mol Cell* **50**: 223-235.

Poser I, Sarov M, Hutchins JR, Heriche JK, Toyoda Y, Pozniakovsky A, Weigl D, Nitzsche A, Hegemann B, Bird AW et al. 2008. BAC TransgeneOmics: a high-throughput method for exploration of protein function in mammals. *Nat Methods* **5**: 409-415.

Sanford JR, Coutinho P, Hackett JA, Wang X, Ranahan W, Caceres JF. 2008. Identification of nuclear and cytoplasmic mRNA targets for the shuttling protein SF2/ASF. *PLoS One* **3**: e3369.

Surendranath V, Theis M, Habermann BH, Buchholz F. 2013. Designing efficient and specific endoribonuclease-prepared siRNAs. *Methods Mol Biol* **942**: 193-204.

Theis M, Buchholz F. 2010. MISSION esiRNA for RNAi screening in mammalian cells. *J Vis Exp* **39**: pii:2008.

Trapnell C, Hendrickson DG, Sauvageau M, Goff L, Rinn JL, Pachter L. 2013. Differential analysis of gene regulation at transcript resolution with RNA-seq. *Nat Biotechnol* **31**: 46-53.

Wang Z, Kayikci M, Briese M, Zarnack K, Luscombe NM, Rot G, Zupan B, Curk T, Ule J. 2010. iCLIP predicts the dual splicing effects of TIA-RNA interactions. *PLoS Biol* **8**: e1000530.

Yeo GW, Coufal NG, Liang TY, Peng GE, Fu XD, Gage FH. 2009. An RNA code for the FOX2 splicing regulator revealed by mapping RNA-protein interactions in stem cells. *Nat Struct Mol Biol* **16**: 130-137.

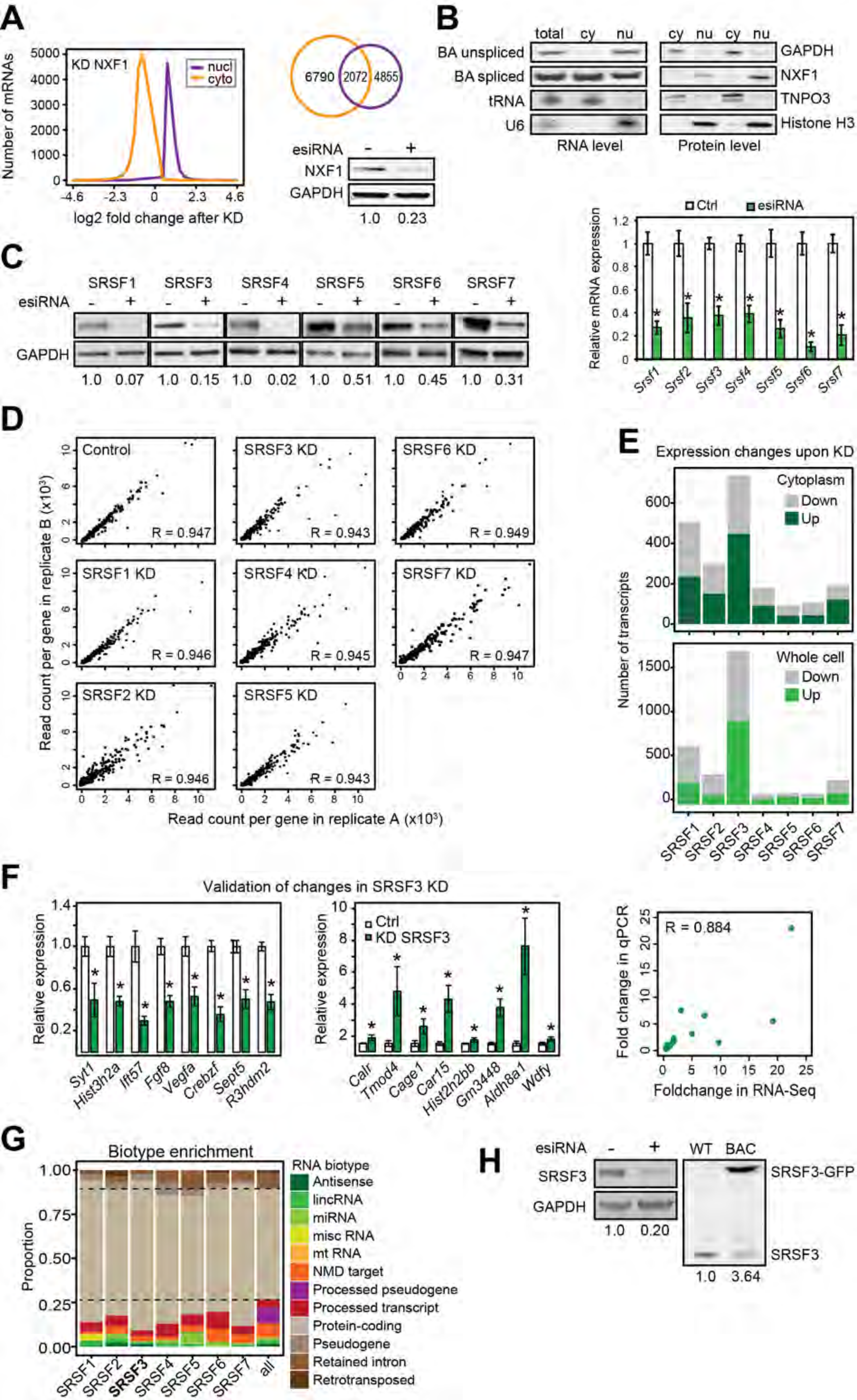
A
KD NXF1
nucl
cyto
Number of mRNAs
log2 fold change after KD
6790
2072
4855
esiRNA
NXF1
GAPDH
1.0
0.23
B
total
cy
nu
BA unspliced
BA spliced
tRNA
U6
RNA level
GAPDH
NXF1
TNPO3
Histone H3
Protein level
C
SRSF1
SRSF3
SRSF4
SRSF5
SRSF6
SRSF7
esiRNA
GAPDH
1.0 0.07 1.0 0.15 1.0 0.02 1.0 0.51 1.0 0.45 1.0 0.31
Ctrl
esiRNA
Relative mRNA expression
Srsf1 Srsf2 Srsf3 Srsf4 Srsf5 Srsf6 Srsf7
D
Control R = 0.947
SRSF3 KD R = 0.943
SRSF6 KD R = 0.949
SRSF1 KD R = 0.946
SRSF4 KD R = 0.945
SRSF7 KD R = 0.947
SRSF2 KD R = 0.946
SRSF5 KD R = 0.943
Read count per gene in replicate B (x10³)
Read count per gene in replicate A (x10³)
E
Expression changes upon KD
Cytoplasm
Down
Up
Whole cell
Down
Up
Number of transcripts
SRSF1 SRSF2 SRSF3 SRSF4 SRSF5 SRSF6 SRSF7
F
Validation of changes in SRSF3 KD
Relative expression
Syt1 Hist3h2a Ift57 Fgf8 Vegfa Crebzf Sept5 R3hdm2
Ctrl
KD SRSF3
Calr Tmod4 Cage1 Car15 Hist2h2bb Gm3448 Aldh8a1 Wdfy
R = 0.884
Fold change in qPCR
Foldchange in RNA-Seq
G
Biotype enrichment
Proportion
SRSF1 SRSF2 SRSF3 SRSF4 SRSF5 SRSF6 SRSF7 all
RNA biotype
Antisense
lincRNA
miRNA
misc RNA
mt RNA
NMD target
Processed pseudogene
Processed transcript
Protein-coding
Pseudogene
Retained intron
Retrotransposed
H
esiRNA
SRSF3
GAPDH
1.0
0.20
WT
BAC
SRSF3-GFP
SRSF3
1.0
3.64

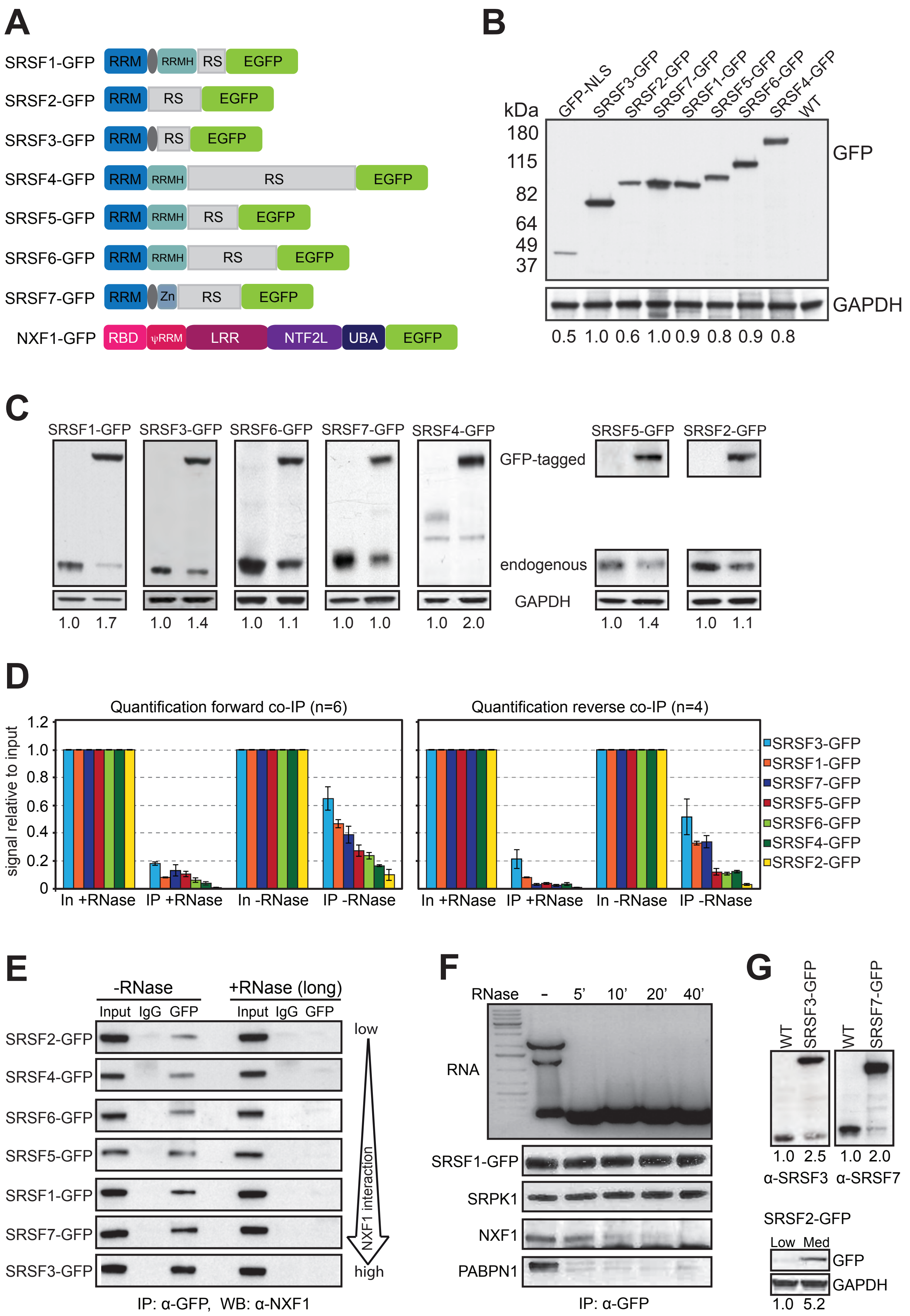
A
SRSF1-GFP RRM RRMH RS EGFP
SRSF2-GFP RRM RS EGFP
SRSF3-GFP RRM RS EGFP
SRSF4-GFP RRM RRMH RS EGFP
SRSF5-GFP RRM RRMH RS EGFP
SRSF6-GFP RRM RRMH RS EGFP
SRSF7-GFP RRM Zn RS EGFP
NXF1-GFP RBD ψRRM LRR NTF2L UBA EGFP
B
GFP-NLS SRSF3-GFP SRSF2-GFP SRSF7-GFP SRSF1-GFP SRSF5-GFP SRSF6-GFP SRSF4-GFP WT
kDa 180 115 82 64 49 37
GFP
GAPDH
0.5 1.0 0.6 1.0 0.9 0.8 0.9 0.8
C
SRSF1-GFP SRSF3-GFP SRSF6-GFP SRSF7-GFP SRSF4-GFP SRSF5-GFP SRSF2-GFP
GFP-tagged
endogenous
GAPDH
1.0 1.7 1.0 1.4 1.0 1.1 1.0 1.0 1.0 2.0 1.0 1.4 1.0 1.1
D
Quantification forward co-IP (n=6)
Quantification reverse co-IP (n=4)
signal relative to input
1.2 1.0 0.8 0.6 0.4 0.2 0
In +RNase IP +RNase In -RNase IP -RNase
SRSF3-GFP SRSF1-GFP SRSF7-GFP SRSF5-GFP SRSF6-GFP SRSF4-GFP SRSF2-GFP
E
-RNase +RNase (long)
Input IgG GFP Input IgG GFP
SRSF2-GFP SRSF4-GFP SRSF6-GFP SRSF5-GFP SRSF1-GFP SRSF7-GFP SRSF3-GFP
low
NXF1 interaction
high
IP: α-GFP, WB: α-NXF1
F
RNase - 5' 10' 20' 40'
RNA
SRSF1-GFP
SRPK1
NXF1
PABPN1
IP: α-GFP
G
WT SRSF3-GFP WT SRSF7-GFP
1.0 2.5 1.0 2.0
α-SRSF3 α-SRSF7
SRSF2-GFP
Low Med
GFP
GAPDH
1.0 5.2

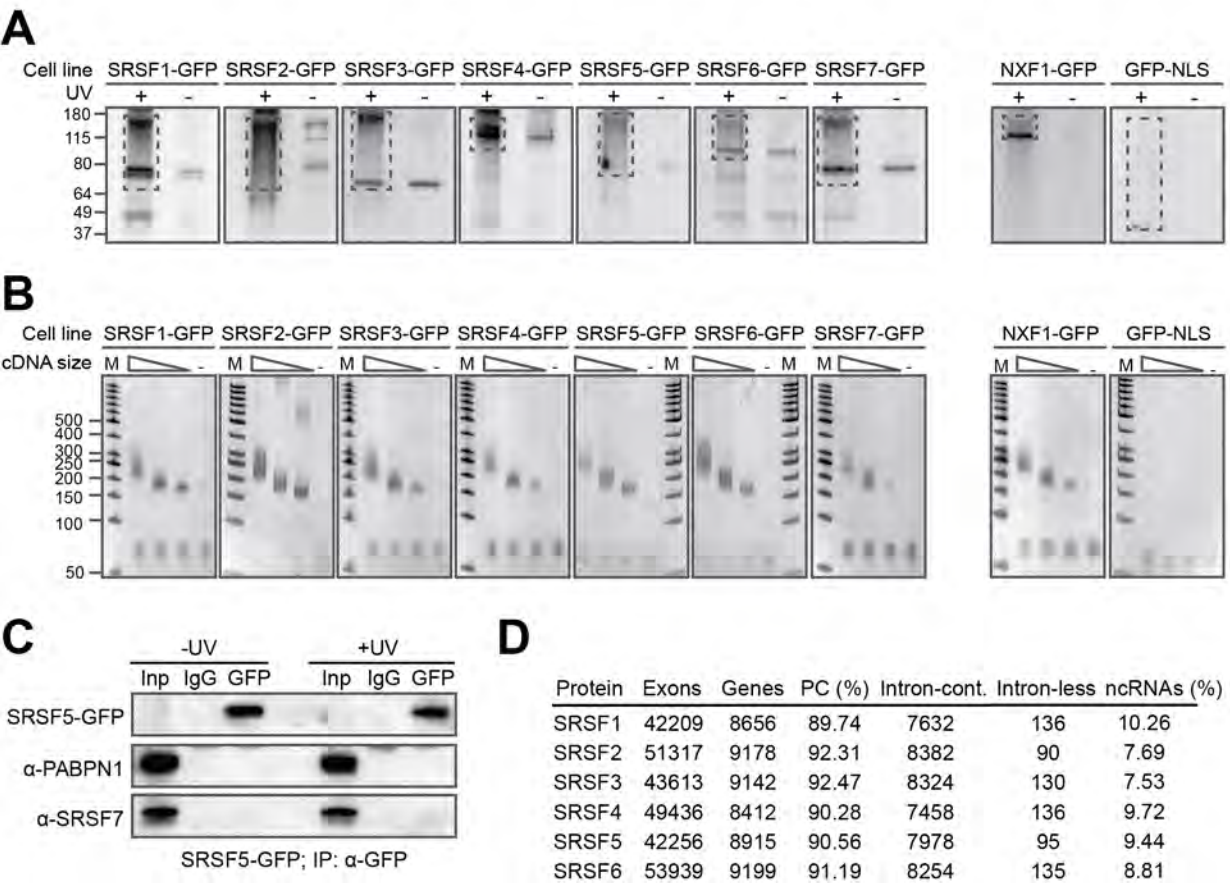

D

Protein	Exons	Genes	PC (%)	Intron-cont.	Intron-less	ncRNAs (%)
SRSF1	42209	8656	89.74	7632	136	10.26
SRSF2	51317	9178	92.31	8382	90	7.69
SRSF3	43613	9142	92.47	8324	130	7.53
SRSF4	49436	8412	90.28	7458	136	9.72
SRSF5	42256	8915	90.56	7978	95	9.44
SRSF6	53939	9199	91.19	8254	135	8.81
SRSF7	48253	8840	91.05	7906	143	8.95
NXF1	48253	5781	85.19	4814	111	14.81

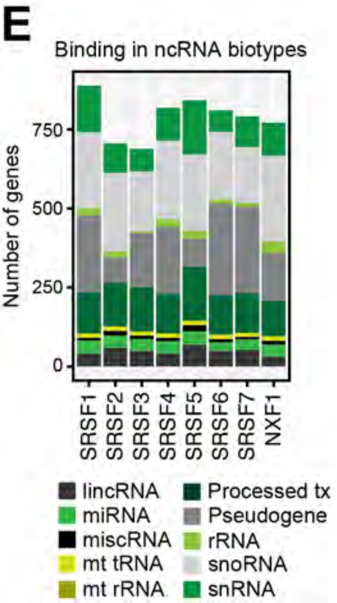

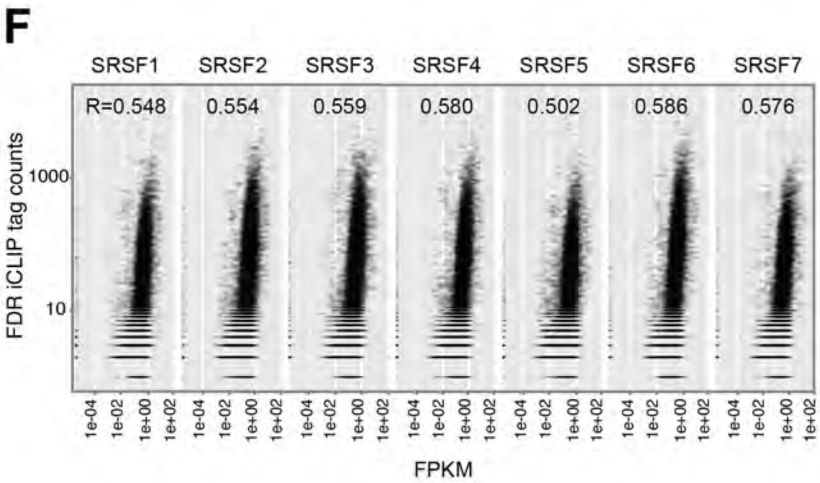

GENESDEV/2015/276477_Müller-McNicoll_Fig. S4

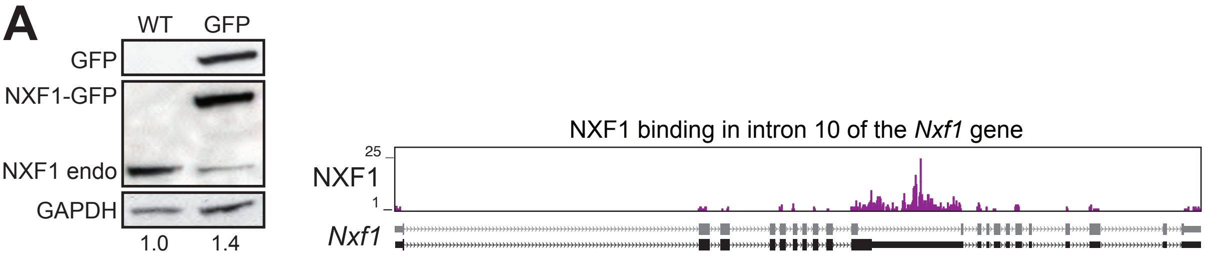

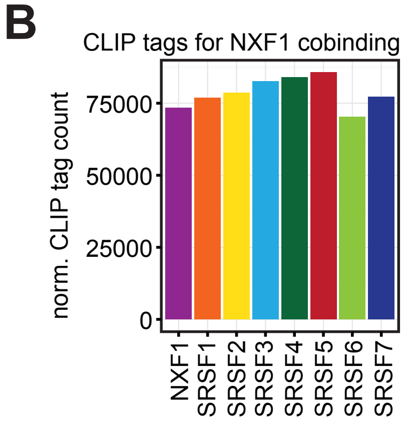

D

Percent cobound exons of all bound exons

Protein	SRSF1	SRSF2	SRSF3	SRSF4	SRSF5	SRSF6	SRSF7
SRSF1	--	84.66	71.42	84.67	72.25	89.29	81.81
SRSF2	69.64	--	70.43	80.25	73.71	87.63	77.95
SRSF3	69.12	82.87	--	80.40	71.73	87.46	82.20
SRSF4	72.30	83.30	70.93	--	72.29	**89.75**	80.45
SRSF5	72.17	**89.51**	74.03	84.57	--	86.27	78.83
SRSF6	69.87	83.37	70.71	82.25	67.59	--	77.44
SRSF7	71.56	82.90	74.30	82.43	69.03	86.57	--

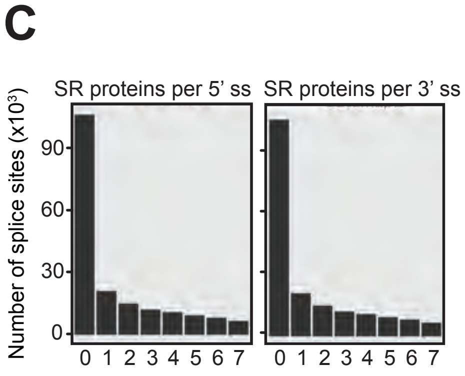

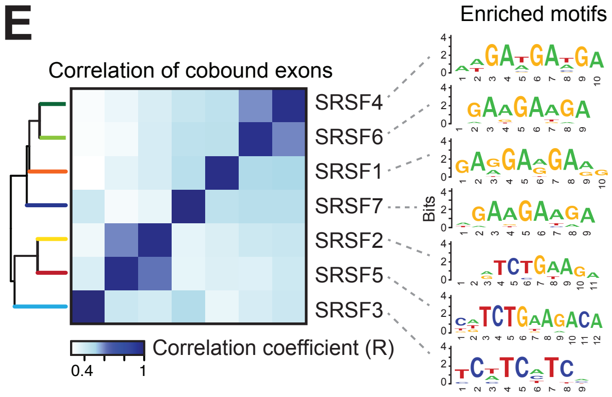

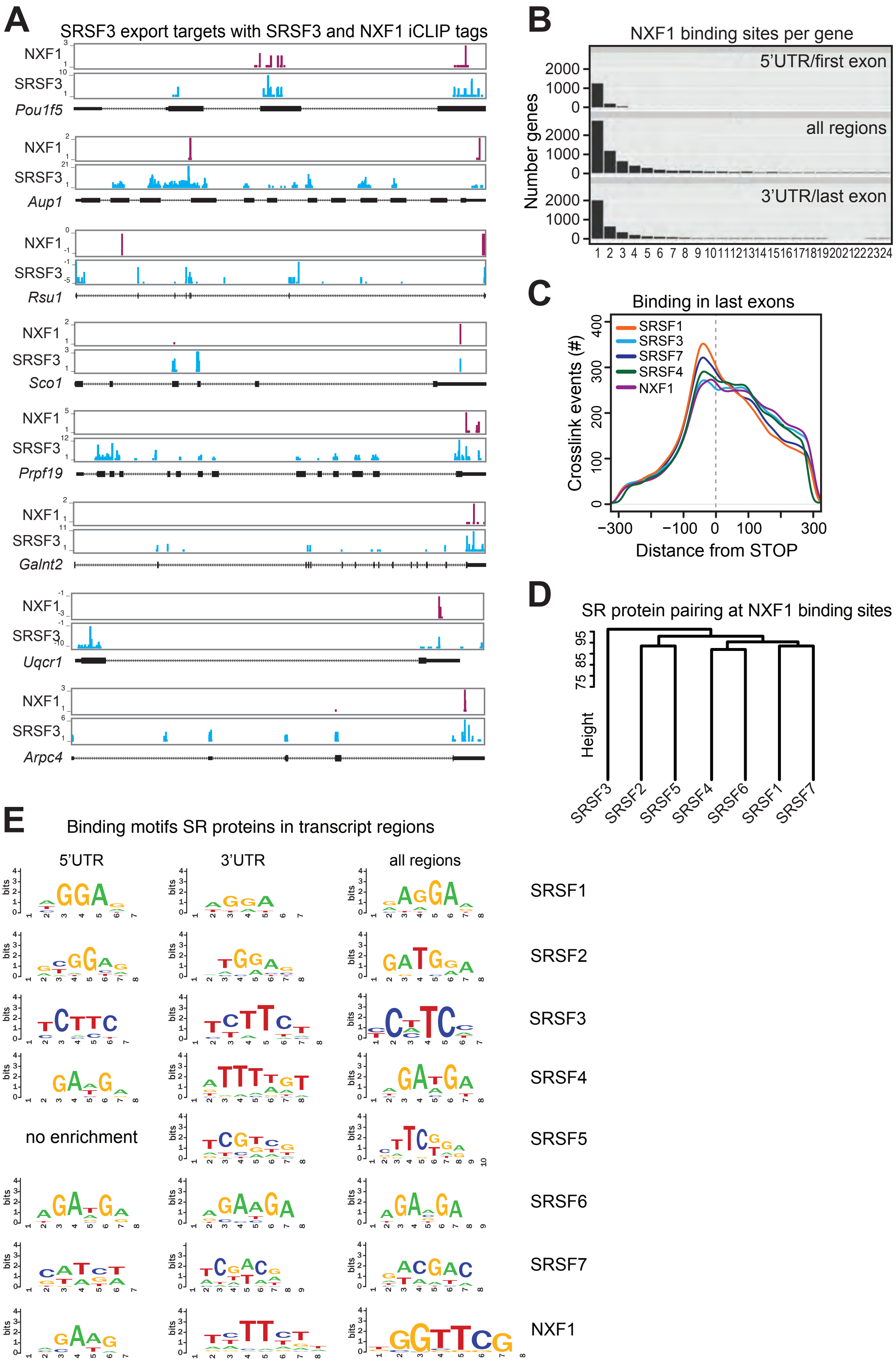
A
SRSF3 export targets with SRSF3 and NXF1 iCLIP tags
NXF1
SRSF3
Pou1f5
Aup1
Rsu1
Sco1
Prpf19
Galnt2
Uqcr1
Arpc4
B
NXF1 binding sites per gene
5'UTR/first exon
all regions
3'UTR/last exon
Number genes
2000
1000
0
1 2 3 4 5 6 7 8 9 10 11 12 13 14 15 16 17 18 19 20 21 22 23 24
C
Binding in last exons
SRSF1
SRSF3
SRSF7
SRSF4
NXF1
Crosslink events (#)
0
100
200
300
400
−300
−100
0
100
300
Distance from STOP
D
SR protein pairing at NXF1 binding sites
75 85 95
Height
SRSF3
SRSF2
SRSF5
SRSF4
SRSF6
SRSF1
SRSF7
E
Binding motifs SR proteins in transcript regions
5'UTR
3'UTR
all regions
SRSF1
SRSF2
SRSF3
SRSF4
no enrichment
SRSF5
SRSF6
SRSF7
NXF1
bits

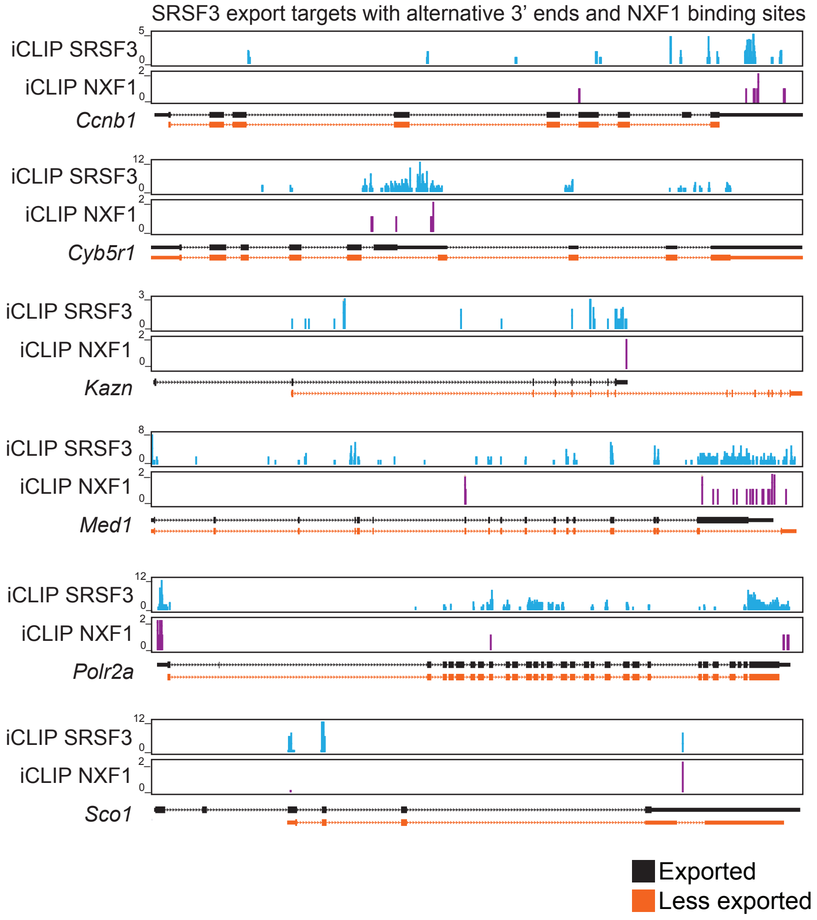
SRSF3 export targets with alternative 3' ends and NXF1 binding sites
iCLIP SRSF3
iCLIP NXF1
Ccnb1
Cyb5r1
Kazn
Med1
Polr2a
Sco1
Exported
Less exported
